# Supplementary material for: Molecular-resolution imaging of ice crystallized from liquid water by cryogenic liquid-cell TEM
Source: Nat Commun. 2025 Sep 25;16:8342. doi: 10.1038/s41467-025-62451-0 (PMC12462463; doi:10.1038/s41467-025-62451-0)
Supplement: Supplementary file 1 — Supplementary Information [file 41467_2025_62451_MOESM1_ESM.pdf]

Supplementary Information

Molecular-resolution imaging of ice crystallized from liquid water by cryogenic liquid-cell TEM

Jingshan S. Du[1], Suvo Banik[2,3], Henry Chan[2], Birk Fritsch[4], Ying Xia[5], Ajay S. Karakoti[1], Andreas Hutzler[4], Subramanian K. R. S. Sankaranarayanan[2,3], James J. De Yoreo[1,5]*

[1]Physical Sciences Division, Pacific Northwest National Laboratory, Richland, WA 99352, United States
[2]Center for Nanoscale Materials, Argonne National Laboratory, Lemont, IL 60439, United States
[3]Department of Mechanical and Industrial Engineering, University of Illinois, Chicago, IL 60607, United States
[4]Helmholtz Institute Erlangen-Nürnberg for Renewable Energy (IEK-11), Forschungszentrum Jülich GmbH, 91058 Erlangen, Germany
[5]Department of Materials Science and Engineering, University of Washington, Seattle, WA 98195, United States

*Correspondence should be addressed to james.deyoreo@pnnl.gov (J.D.Y.)

This PDF file contains:

Supplementary Note 1. Additional Microscopy and Characterization Results
Supplementary Note 2. Additional Details for Molecular Dynamics Simulation
Supplementary Note 3. Additional Details for Theoretical Calculations
Supplementary Tables 1–9
Supplementary Figures 1–42
Supplementary References

Supplementary Note 1. Additional Microscopy and Characterization Results

Supplementary Note 1.1. Carbon membrane characteristics

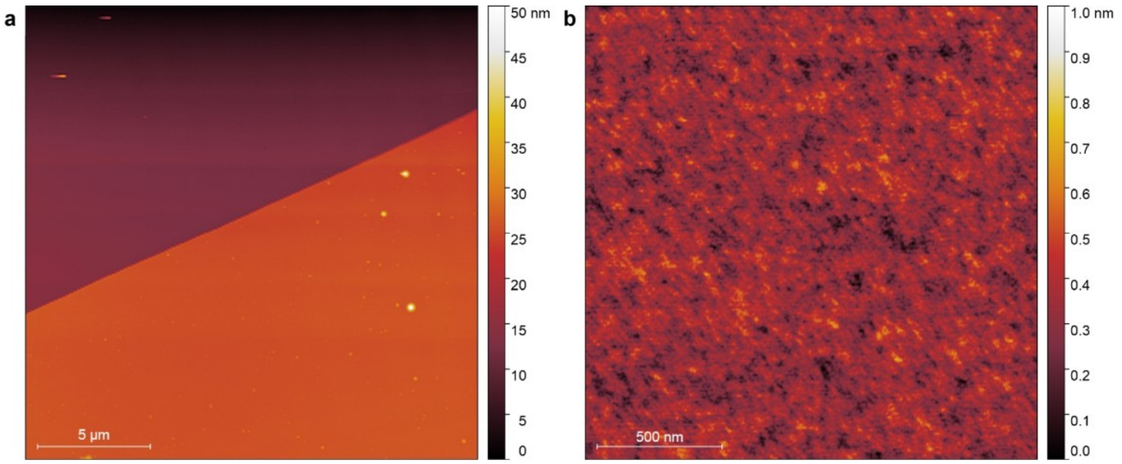

Supplementary Fig. 1. Characterization of the amorphous carbon membrane used in this study by AFM. a, Height image of a broken edge of the thin film for thickness determination: terrace-fitted step height = 12.0 nm. **b,** Height image of the film surface for roughness measurement: RMS roughness S_q = 83.4 pm and mean roughness S_a = 66.3 pm.

We studied the surface chemical characteristics of the carbon membrane using X-ray photoelectron spectroscopy (XPS), as shown in Supplementary Fig. 2. The C 1*s* core level spectrum was deconvolved to assess the relative contributions of various carbon environments. The peak deconvolution showed contributions from carbon in both sp^2 and sp^3 environments from the amorphous carbon. The sp^2 carbon contribution was found to be 55% which agrees with the *D* parameter value assessed from the C KVV Auger emission[1]. In addition to the hydrocarbon, a peak corresponding to C-O at 286.8 eV was also present and its relative concentration of 7% matches well with the 6.3% oxygen present in the overall sample. The other two peaks were assigned to the π–π^* shake-up structure at 291.2 eV, while a secondary peak was fitted to account for the asymmetry in the sp^2 carbon 1*s* peak.

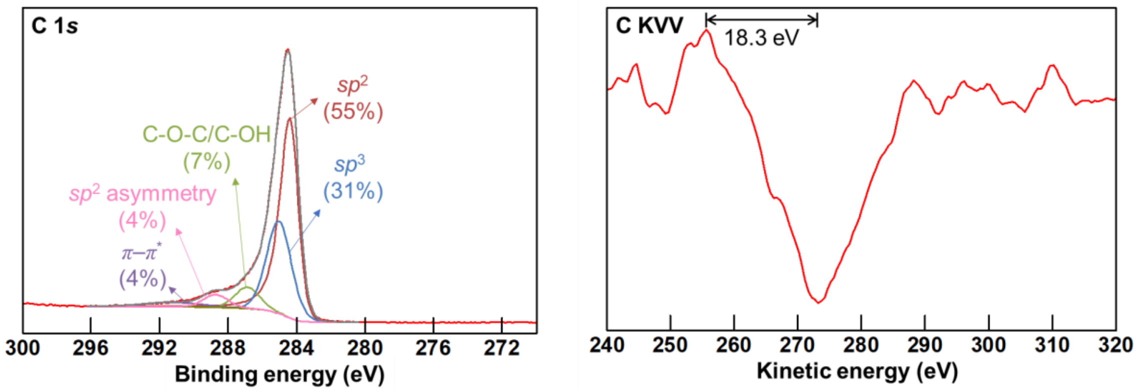

Supplementary Fig. 2. XPS analysis of the carbon membrane.

The water contact angle on the carbon membrane (supported on the Cu grid) was measured to be 63.0° ± 1.8° through drop-shape fitting[2] (Supplementary Fig. 3; independently measured on three grids). This result is similar to that on freshly exfoliated highly oriented pyrolytic graphite (HOPG) in air[3] and is consistent with the presence of mild oxidation and hydrocarbon adsorption on the membrane surface.

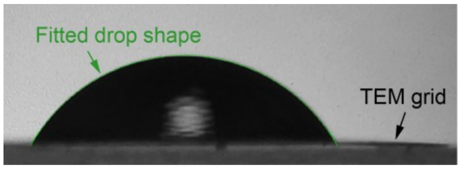

Supplementary Fig. 3. Water contact angle measurement on carbon membrane.

Supplementary Note 1.2. Sample crystallography

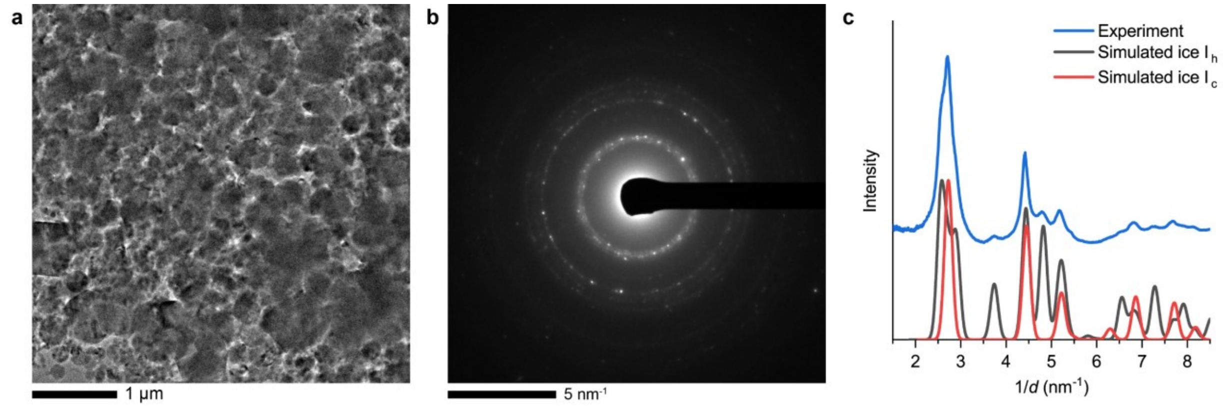

Supplementary Fig. 4. Condensed ice crystallites made by exposing the cryogenic sample (< −180 °C) in the air for a few seconds. a, TEM image. **b,** SAED pattern. **c,** Radial profile of the experimental SAED compared to the simulated patterns assuming ice I_h and I_c.

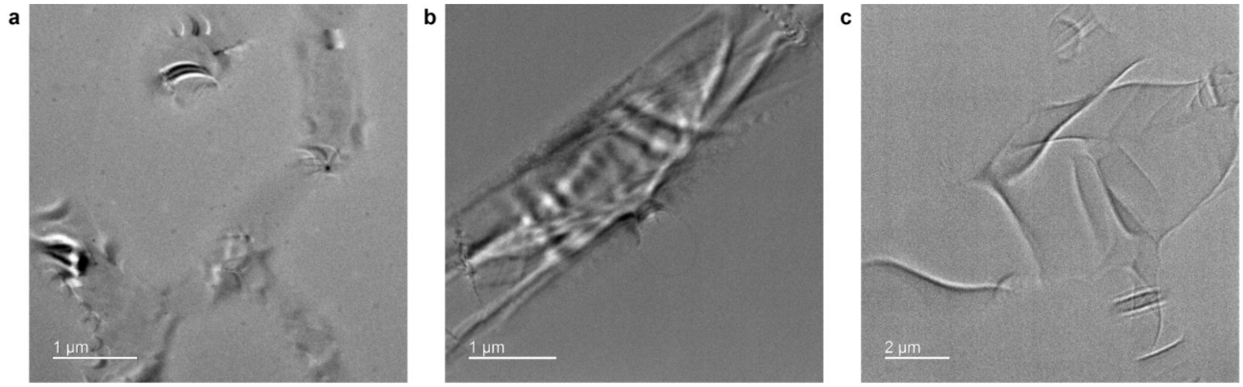

Supplementary Fig. 5. Wide-angle TEM images showing encapsulated ice crystals along different zone axes (where diffraction patterns and HRTEM images in this section were obtained). a, [0001]. **b,** [**$11\bar{2}3$**]. **c,** [**$10\bar{1}0$**].

a b

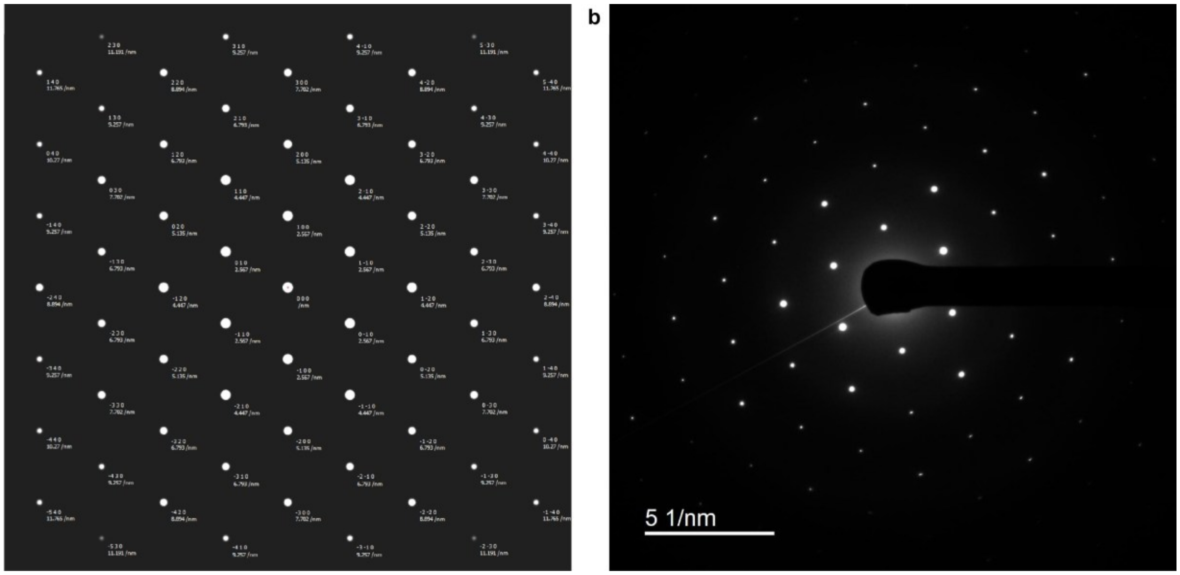

Supplementary Fig. 6. SAED of ice I_h along the [0001] zone axis. a, Dynamically simulated SAED. **b,** Experimental SAED.

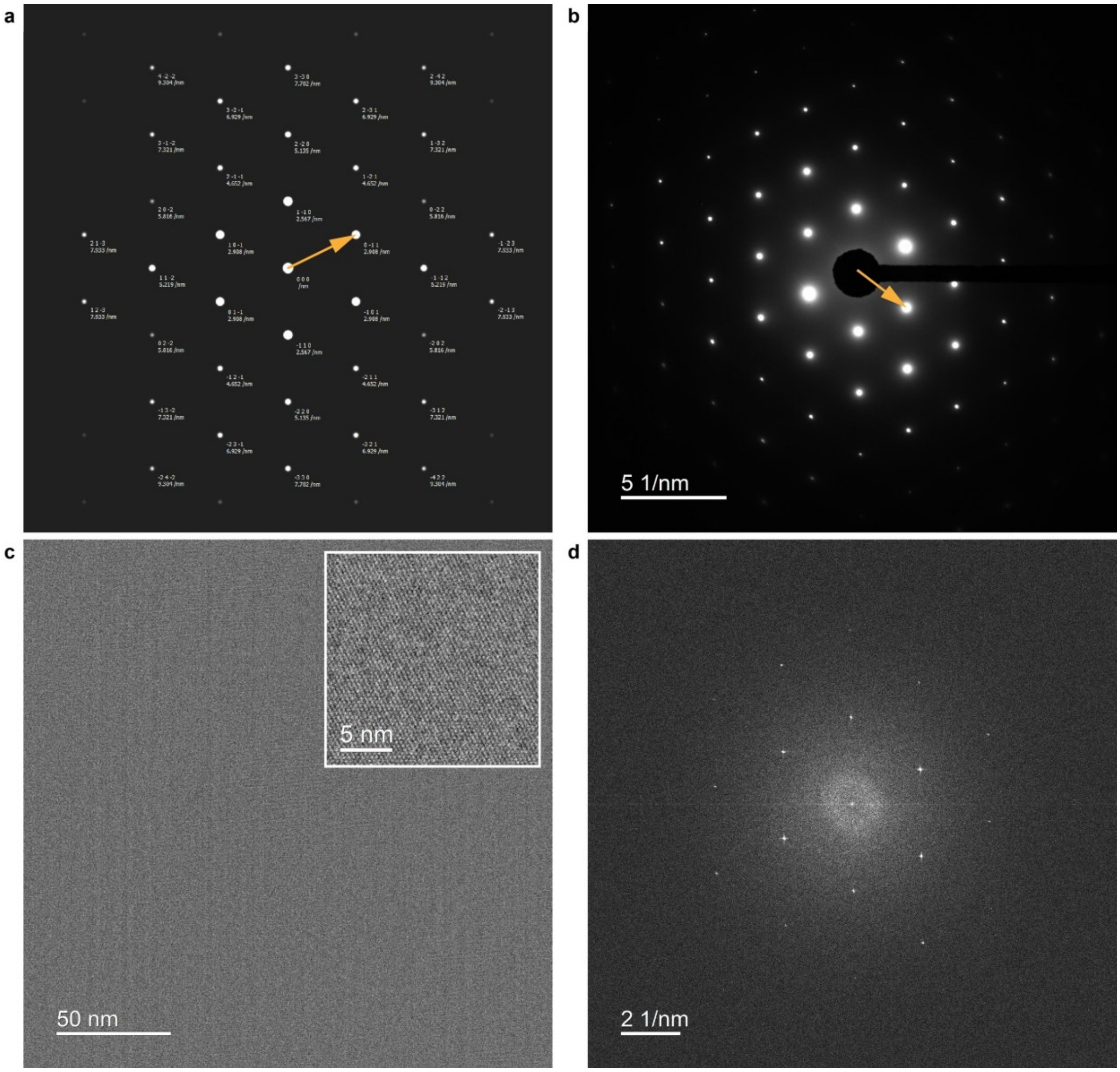

Supplementary Fig. 7. SAED and HRTEM of ice I_h along the [11$\bar{2}$3] zone axis (Miller: [111]). a, Dynamically simulated SAED. **b,** Experimental SAED. **c,** ABS-filtered HRTEM image. Inset: enlarged image (see also Fig. 1e). **d,** Fourier transform of unfiltered HRTEM image. Arrows in **a** and **b** indicate the same reflections.

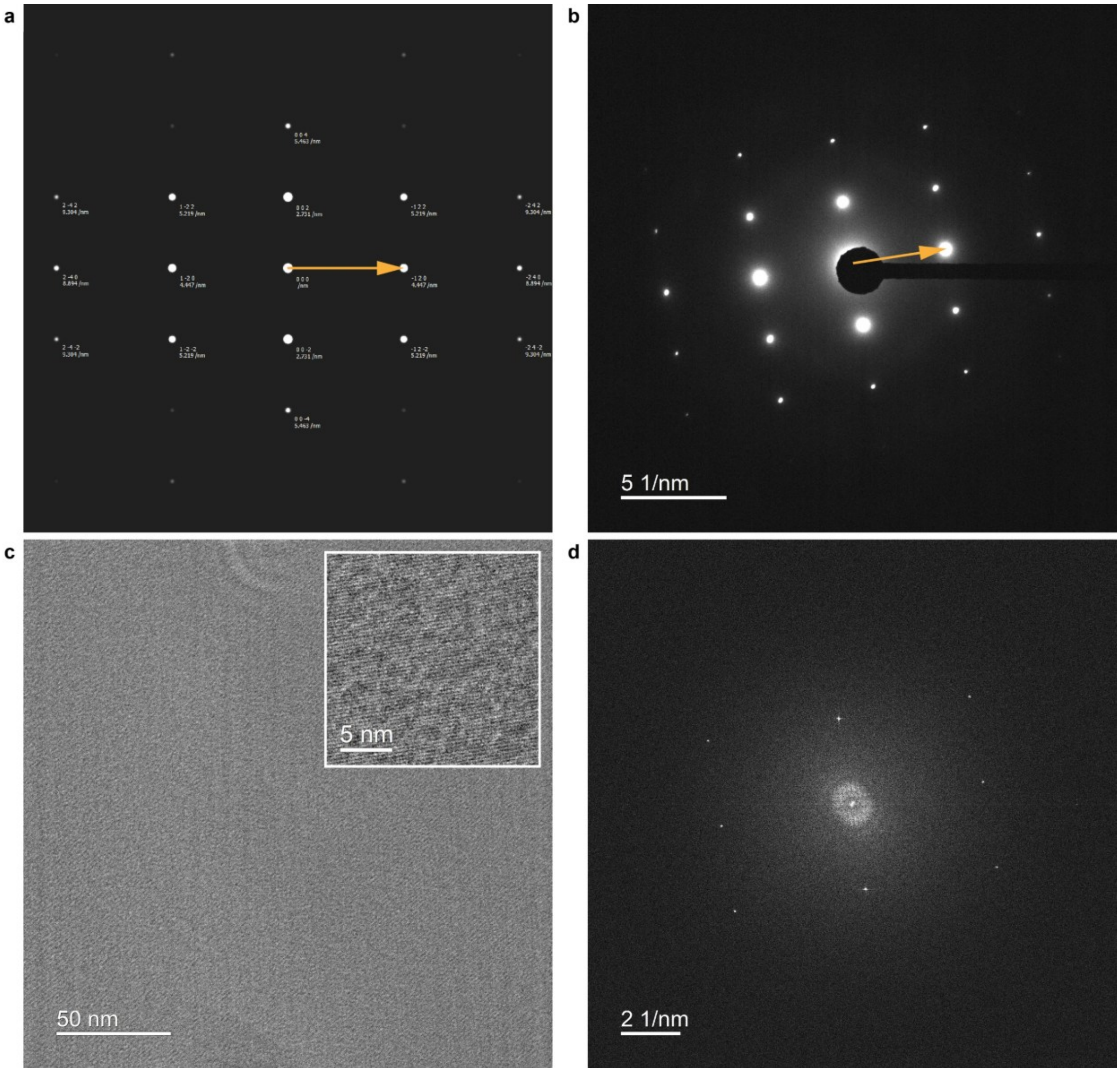

Supplementary Fig. 8. SAED and HRTEM of ice I_h along the $[10\bar{1}0]$ zone axis (Miller: [210]). a, Dynamically simulated SAED. **b,** Experimental SAED. **c,** ABS-filtered HRTEM image. Inset: enlarged image (see also Fig. 1f). **d,** Fourier transform of unfiltered HRTEM image. Arrows in **a** and **b** indicate the same reflections.

Supplementary Note 1.3. Sample thickness and stability

To estimate the thickness of the encapsulated, crystalline ice samples, low-magnification electron energy-loss spectroscopy (EELS) spectral imaging at 300 kV was performed. Here, three randomly picked areas containing large, crystalline ice samples (areas over microns) were imaged by TEM and HAADF (Supplementary Fig. 9a–c). The contours in the TEM image are characteristic of high-quality crystals with thickness gradients and bending. Then, thickness values (h) were calculated, which are inherently relative to the electron mean free path (λ). For electrons at 300 keV in ice, this value is ≈400 nm[4]. As such, the typical thickness near the crystal edges ranges from tens to hundreds of nanometers (Supplementary Fig. 9d), consistent with the desired conditions to form HRTEM phase contrast images.

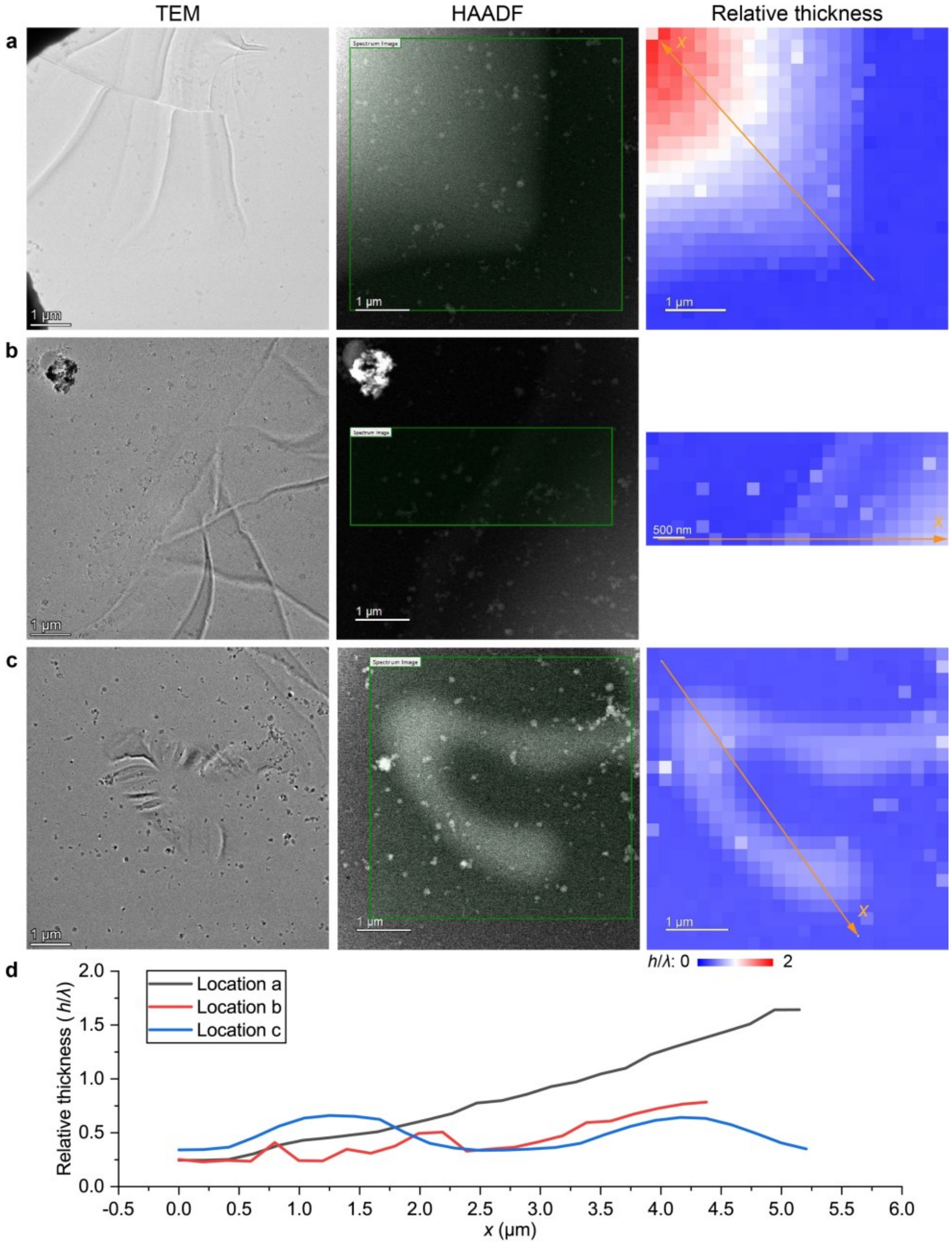

Supplementary Fig. 9. Low-magnification TEM, HAADF, and relative thickness mapping results with EELS for three different sample areas (a–c) and corresponding thickness line profiles (d).

The stability of the encapsulated ice crystals under sustained electron irradiation was investigated by taking time-series HRTEM images at controlled electron flux. Here, two conditions were studied: 60 and 100 e $Å^{-2}$ s^{-1}. In each case, HRTEM images were taken every 10 s for a total duration of 10 min (Supplementary Movies 1 and 2). The samples show high crystallinity after these extended electron irradiation experiments, and high-quality HRTEM images can still be obtained (Supplementary Fig. 10). These results evidence that the membrane-encapsulated ice samples are highly stable under HRTEM conditions, sufficient for extended imaging tasks.

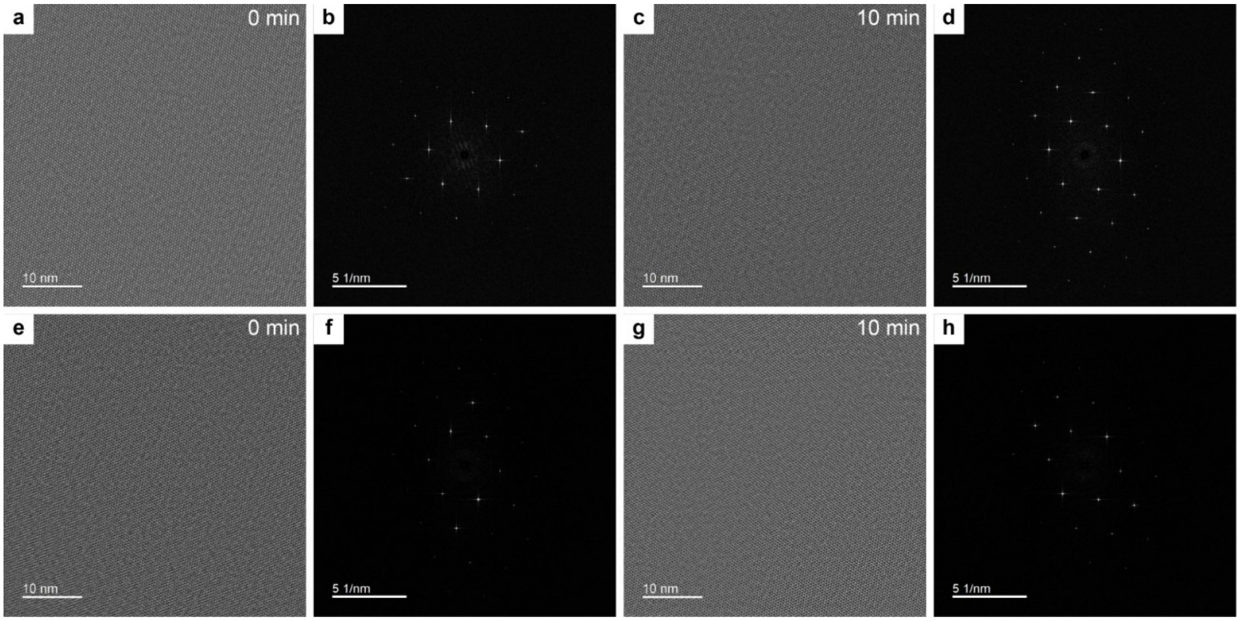

Supplementary Fig. 10. Time-series HRTEM of an ice crystal along the [0001] zone axis under sustained electron irradiation at 60 e $Å^{-2}$ s^{-1} (a–d) and 100 e $Å^{-2}$ s^{-1} (e–h). a,c,e,g, ABS-filtered HRTEM images. **b,d,f,h,** Corresponding Fourier transform.

The thickness variation under the experimental radiolysis conditions for nanobubbles (−70 °C, 25 e $Å^{-2}$ s^{-1}) was further studied by energy-filtered TEM (EFTEM) under flux-controlled electron irradiation. Specifically, relative thickness maps in terms of the electron mean free path were calculated by comparing the unfiltered TEM image and zero-loss peak (ZLP)-EFTEM image (0 ± 5 eV) in the same area (Supplementary Fig. 11a–c). This result shows that the sample thickness is stable on a time scale of 10 min. We observed bubble formation and coarsening near the edge of the crystal while the bulk remained intact.

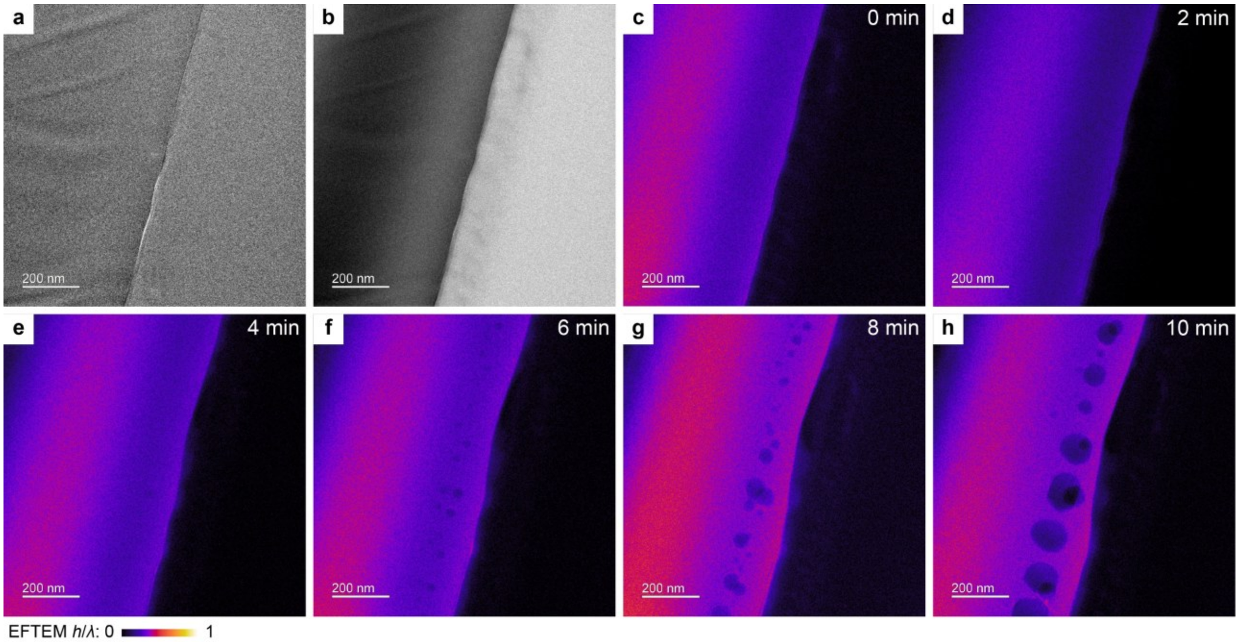

Supplementary Fig. 11. EFTEM for an ice crystal under the radiolysis experimental conditions (−70 °C, 25 e Å^{-2} s^{-1}). a–c, Initial unfiltered TEM (**a**), ZLP-EFTEM (**b**), and calculated thickness map (**c**). **d–h,** Calculated thickness maps with a time interval of 2 min under sustained electron irradiation.

Supplementary Note 1.4. Lattice mapping

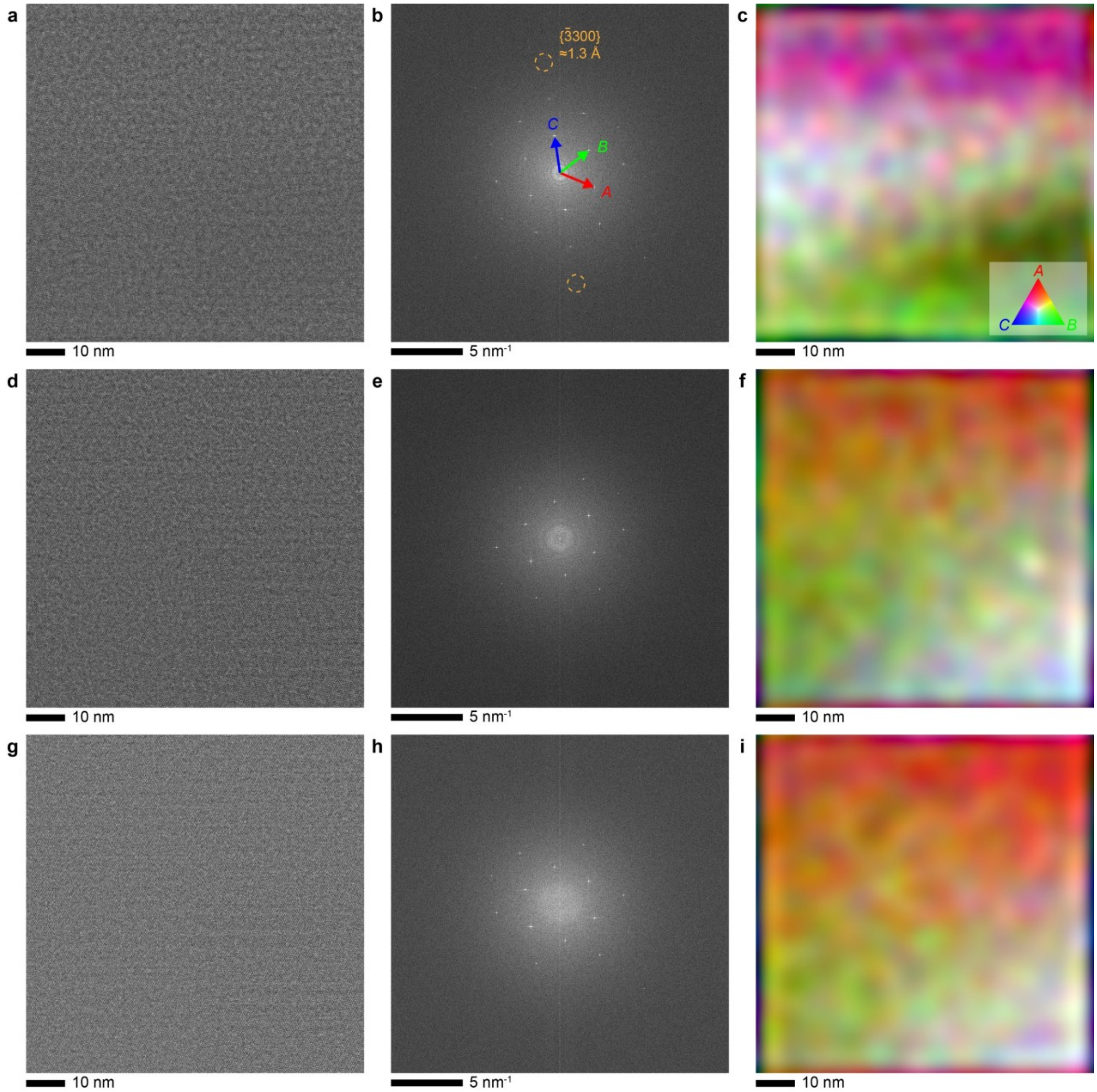

Supplementary Fig. 12. High-resolution transmission electron microscopy (HRTEM) of a continuous hexagonal ice section along the [0001] zone axis with varying defocus (by row). a,d,g, ABS-filtered HRTEM images. **b,e,h,** Fourier transform of unfiltered HRTEM images. **c,f,i,** Lattice amplitude maps. All maps share the color coding specified in **b** and **c**. A negative defocus was applied and reduced toward zero from **a** to **d** and **g**. For case **a**, see also Fig. 1d.

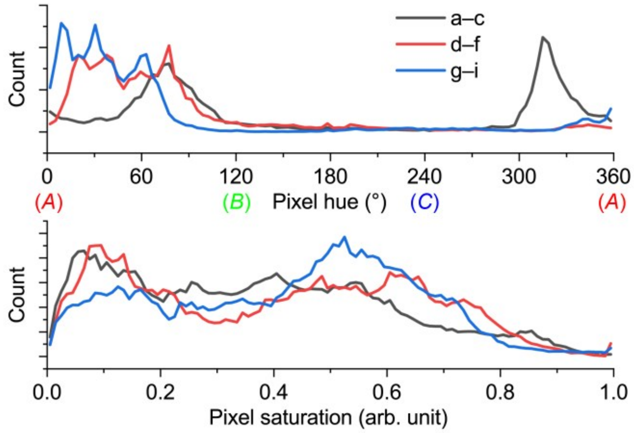

Supplementary Fig. 13. Histograms of pixel hue (upper) and saturation (lower) in the hue-saturation-intensity (HSI) model of images in Supplementary Fig. 12.

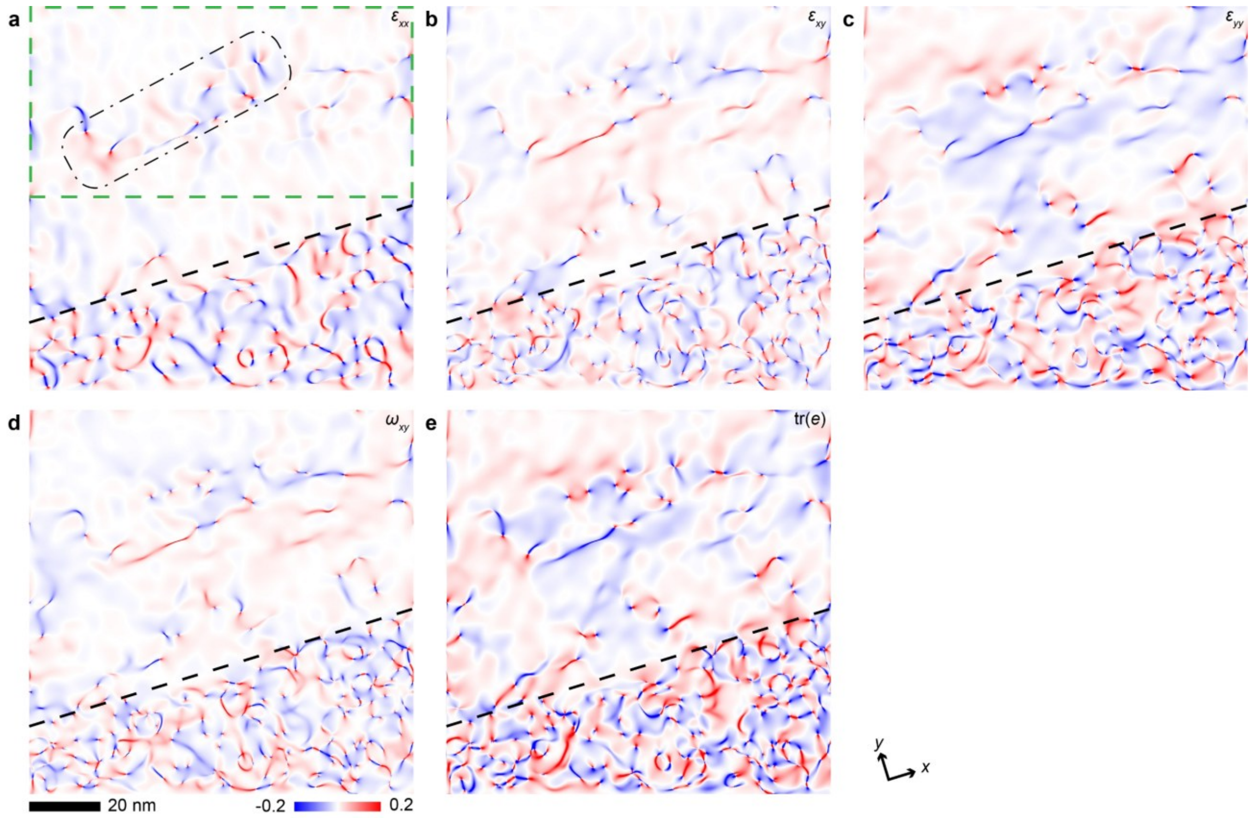

Supplementary Fig. 14. In-plane geometric phase analysis for the defective edge. a, Strain ε_{xx}. **b,** Strain ε_{xy}. **c,** Strain ε_{yy}. **d,** Rotation ω_{xy}. **e,** Dilation tr(e). A green dashed box in **a** indicates the area used to generate the histogram (Fig. 4h). Dot-dashes in **a** indicate a strip-shaped area with concentrated strains in the surroundings.

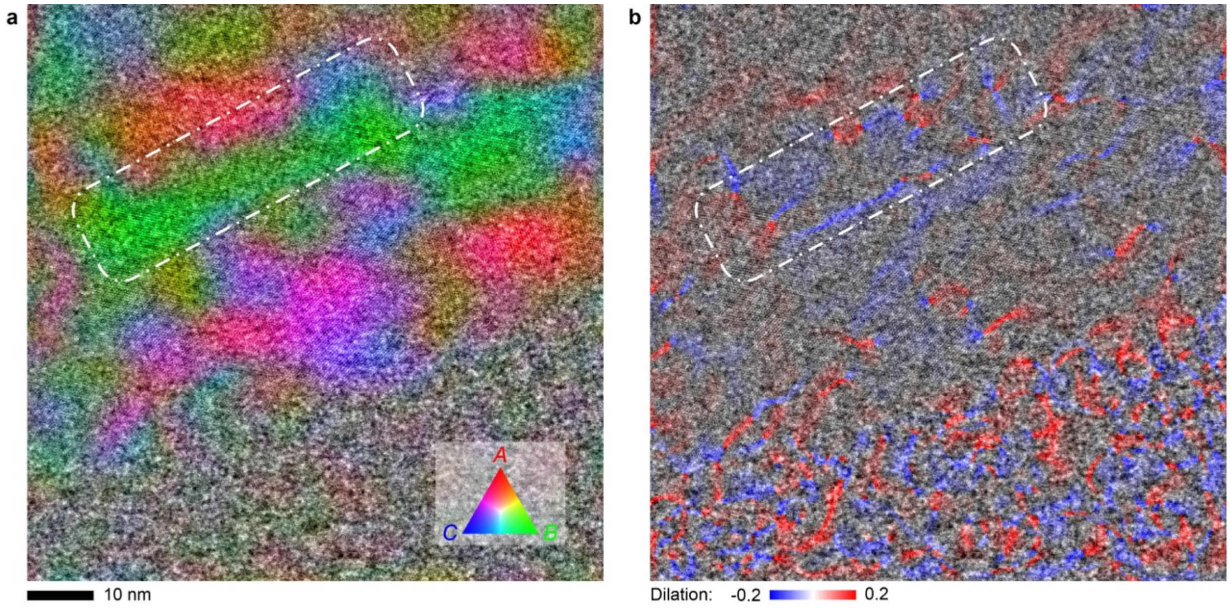

Supplementary Fig. 15. Color-painted ABS-filtered HRTEM images of the defective edge. a, Lattice amplitude. **b,** Lattice dilation. Dot-dashes indicate the same area defined in Supplementary Fig. 14.

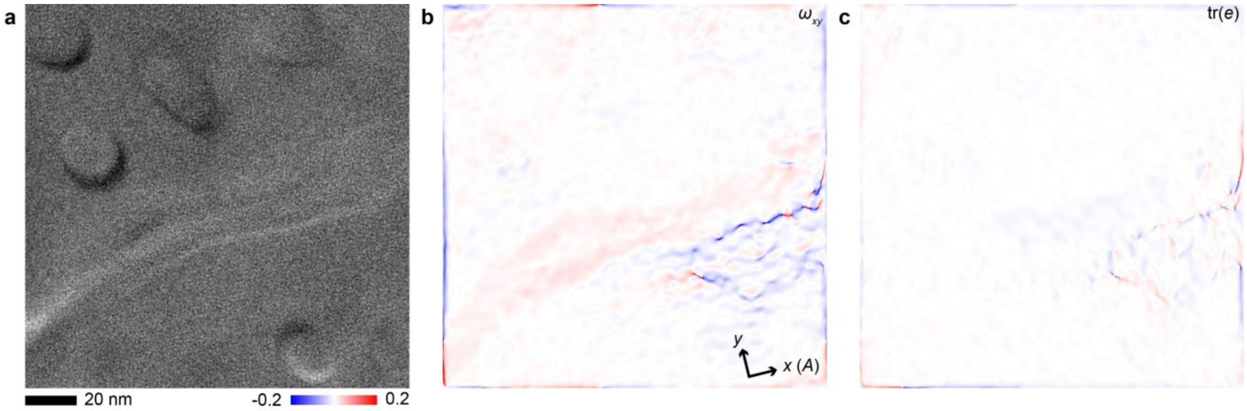

Supplementary Fig. 16. Additional lattice maps for the ice section shown in Fig. 4. a, HRTEM. **b,** Lattice rotation. **c,** Lattice dilation.

Supplementary Note 1.5. *Lattice fringe visibility band*

A quick way to calculate the visibility of a certain lattice fringe from HRTEM images is to evaluate the intersection between the Ewald sphere and the diffraction spot in the reciprocal space. With kinematic approximation, the upper limit of the half angle to keep such an intersection is[5]:

$$\alpha_{\max} = \sin^{-1}\left\{d\frac{\Gamma}{h} + \frac{\lambda}{2d}\left[1 - \left(d\frac{\Gamma}{h}\right)^2\right]\right\}$$

Supplementary Eq. (1)

Here, $d = 0.3895$ nm is the lattice spacing of ice $\{\bar{1}100\}$, Γ is a visibility factor on the order of 1 relevant to the imaging and detection conditions, h is the crystal thickness, $\lambda = 1.97$ pm is the electron beam wavelength at 300 kV. This calculation shows that to observe the ice $\{\bar{1}100\}$ lattices in HRTEM, the maximum tilt angle of the crystals is about 1° to 2° when crystal thickness is 10 to 20 nm (Supplementary Fig. 17).

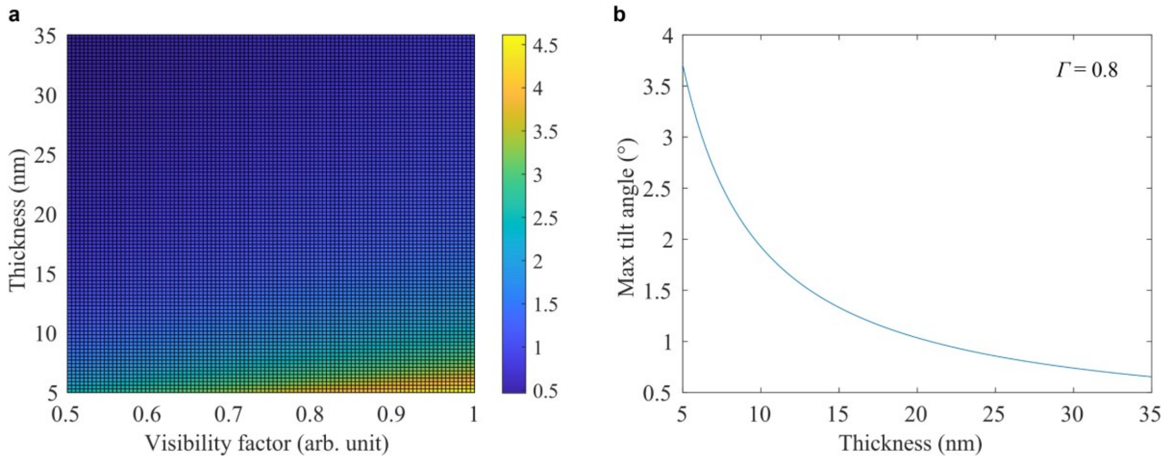

Supplementary Fig. 17. Estimation of max tilt angle (°) for the visibility of ice $\{\bar{1}100\}$ lattice fringes. **a,** Colormap as a function of the visibility factor (Γ) and crystal thickness. **b,** Plot for visibility factor = 0.8.

Supplementary Note 1.6. _Kinematical TEM simulation: low defocus_

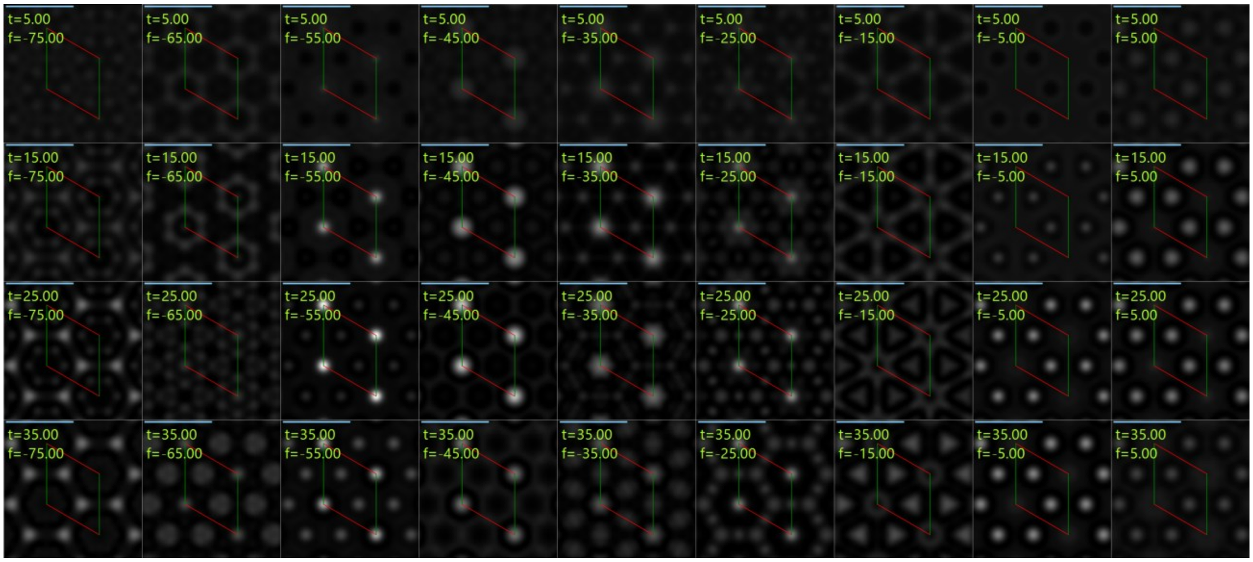

Supplementary Fig. 18. Kinematically simulated HRTEM image matrix of ice I_h along the [0001] zone axis. Thickness (t) varies from 5 to 35 nm (by row). Defocus (f) varies from −75 to +5 nm (by column). Scale bars (cyan): 0.5 nm. A unit cell is outlined in each image. Orange and cyan asterisks note two types of patterns commonly observed in the experiments.

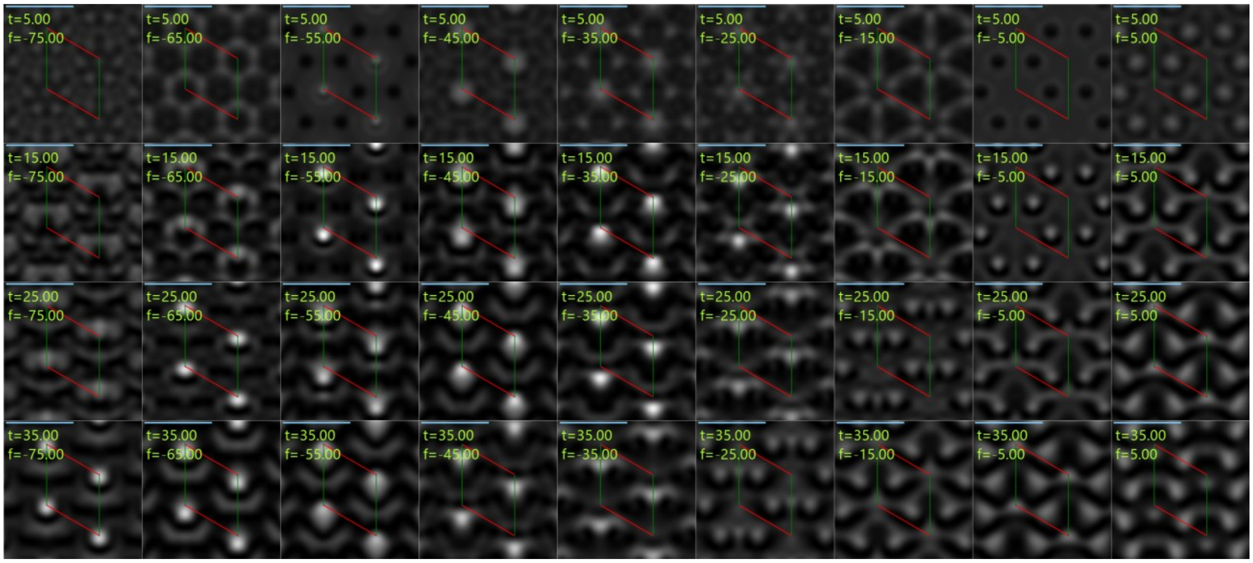

Supplementary Fig. 19. Kinematically simulated HRTEM image matrix of ice I_h along the [0001] zone axis tilted by 0.4° towards the a_2-axis direction (vertical). Thickness (t) varies from 5 to 35 nm (by row). Defocus (f) varies from −75 to +5 nm (by column). Scale bars (cyan): 0.5 nm. A unit cell is outlined in each image.

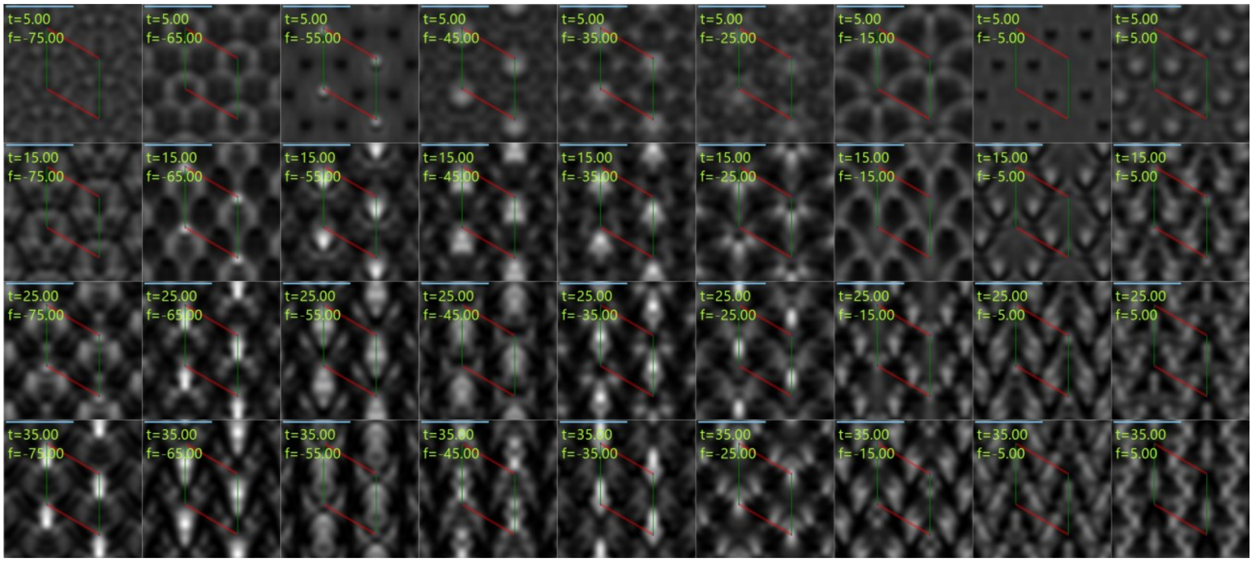

Supplementary Fig. 20. Kinematically simulated HRTEM image matrix of ice I_h along the [0001] zone axis tilted by 0.8° towards the a_2-axis direction (vertical). Thickness (t) varies from 5 to 35 nm (by row). Defocus (f) varies from −75 to +5 nm (by column). Scale bars (cyan): 0.5 nm. A unit cell is outlined in each image.

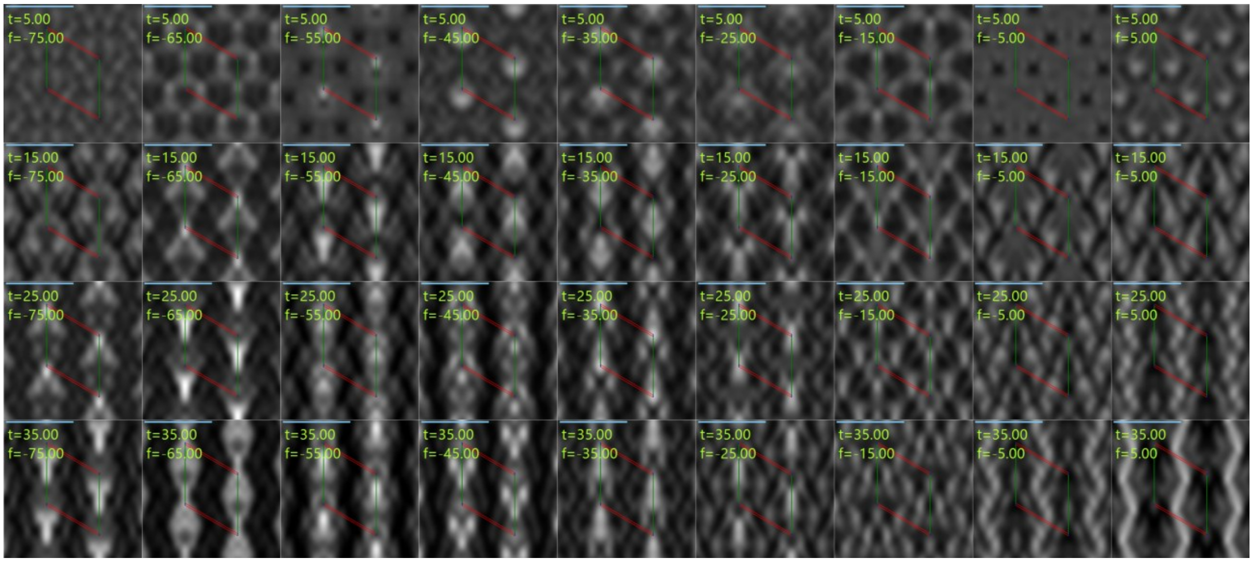

Supplementary Fig. 21. Kinematically simulated HRTEM image matrix of ice I_h along the [0001] zone axis tilted by 1.2° towards the a_2-axis direction (vertical). Thickness (t) varies from 5 to 35 nm (by row). Defocus (f) varies from −75 to +5 nm (by column). Scale bars (cyan): 0.5 nm. A unit cell is outlined in each image.

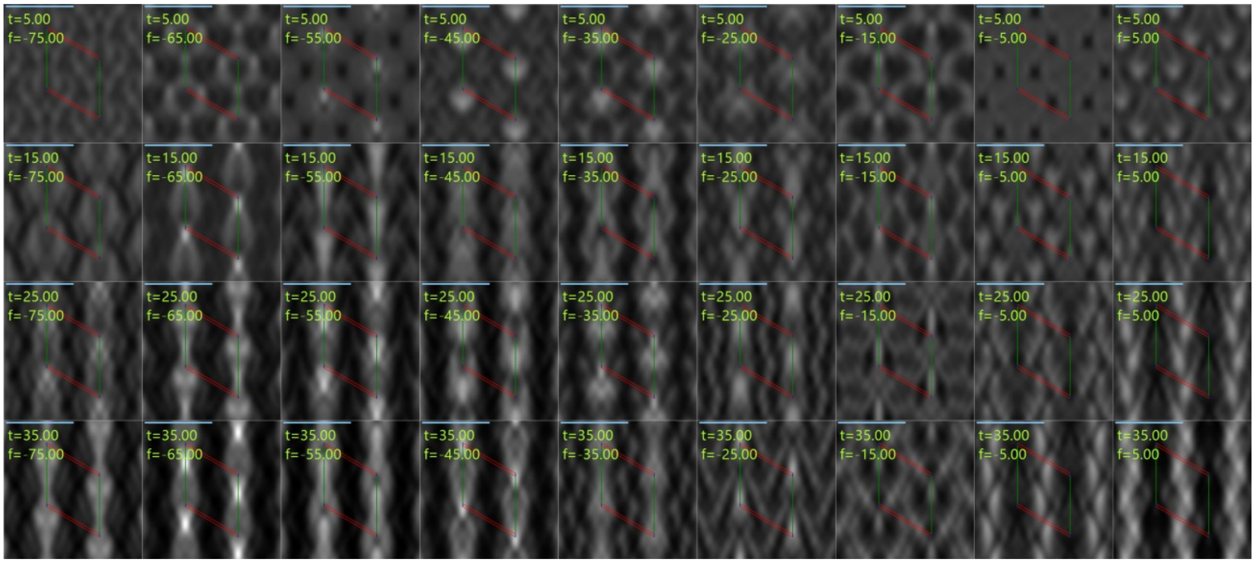

Supplementary Fig. 22. Kinematically simulated HRTEM image matrix of ice I_h along the [0001] zone axis tilted by 1.6° towards the a_2-axis direction (vertical). Thickness (t) varies from 5 to 35 nm (by row). Defocus (f) varies from −75 to +5 nm (by column). Scale bars (cyan): 0.5 nm. A unit cell is outlined in each image.

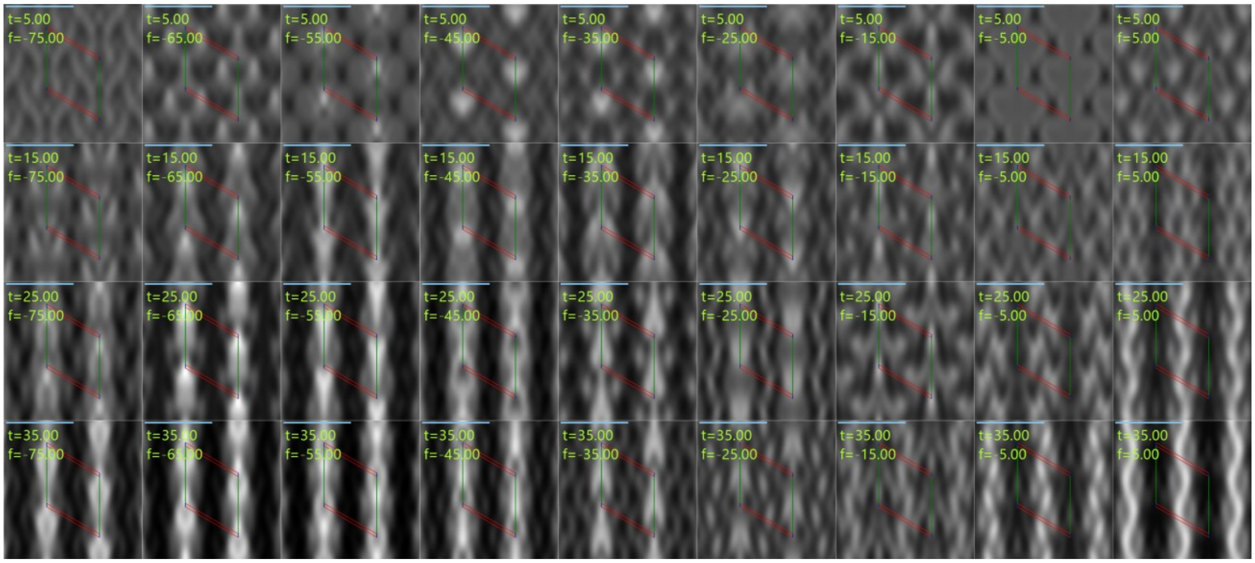

Supplementary Fig. 23. Kinematically simulated HRTEM image matrix of ice I_h along the [0001] zone axis tilted by 2.0° towards the a_2-axis direction (vertical). Thickness (t) varies from 5 to 35 nm (by row). Defocus (f) varies from −75 to +5 nm (by column). Scale bars (cyan): 0.5 nm. A unit cell is outlined in each image.

Supplementary Note 1.7. Kinematical TEM simulation: high defocus

While imaging the highly defective, thin crystal edges (Fig. 2), a slightly larger defocus than what is typically used for materials samples is applied to provide sufficient image contrast from ice. In this specific case, fitting the contrast transfer function of the HRTEM image[6] results in defocus values of −271 and −240 nm. Therefore, we further performed kinematical TEM simulations under high-defocus conditions as below.

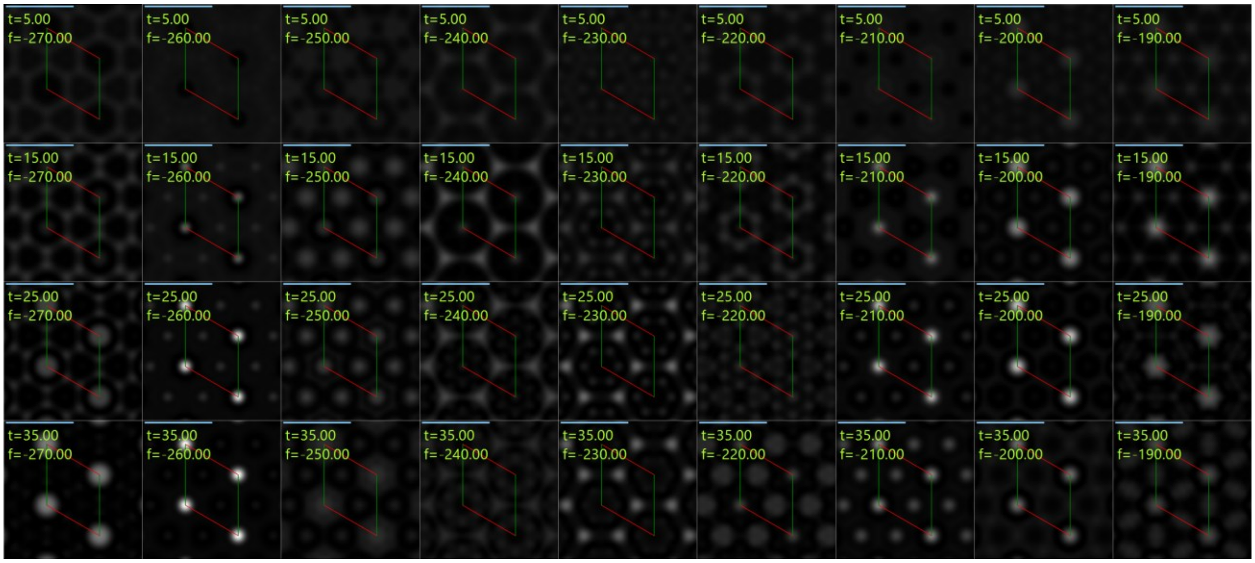

Supplementary Fig. 24. Kinematically simulated HRTEM image matrix with high defocus of ice I_h along the [0001] zone axis. Thickness (t) varies from 5 to 35 nm (by row). Defocus (f) varies from −270 to −190 nm (by column). Scale bars (cyan): 0.5 nm. A unit cell is outlined in each image. Orange asterisks note patterns matching experimental observations.

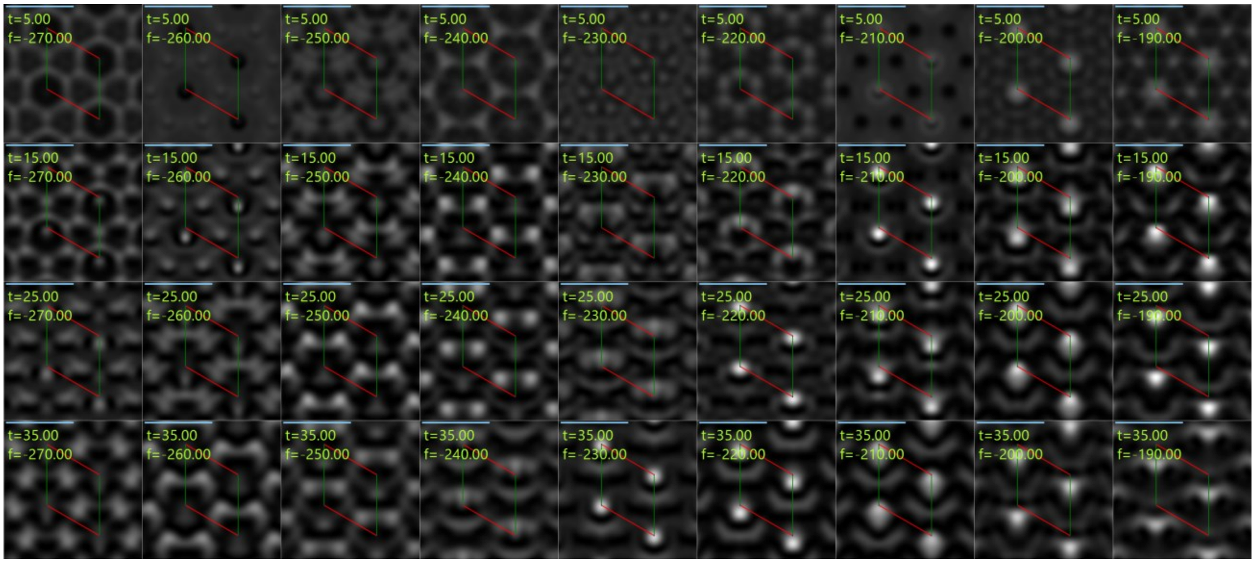

Supplementary Fig. 25. Kinematically simulated HRTEM image matrix with high defocus of ice I_h along the [0001] zone axis tilted by 0.4° towards the a_2-axis direction (vertical). Thickness (t) varies from 5 to 35 nm (by row). Defocus (f) varies from −270 to −190 nm (by column). Scale bars (cyan): 0.5 nm. A unit cell is outlined in each image.

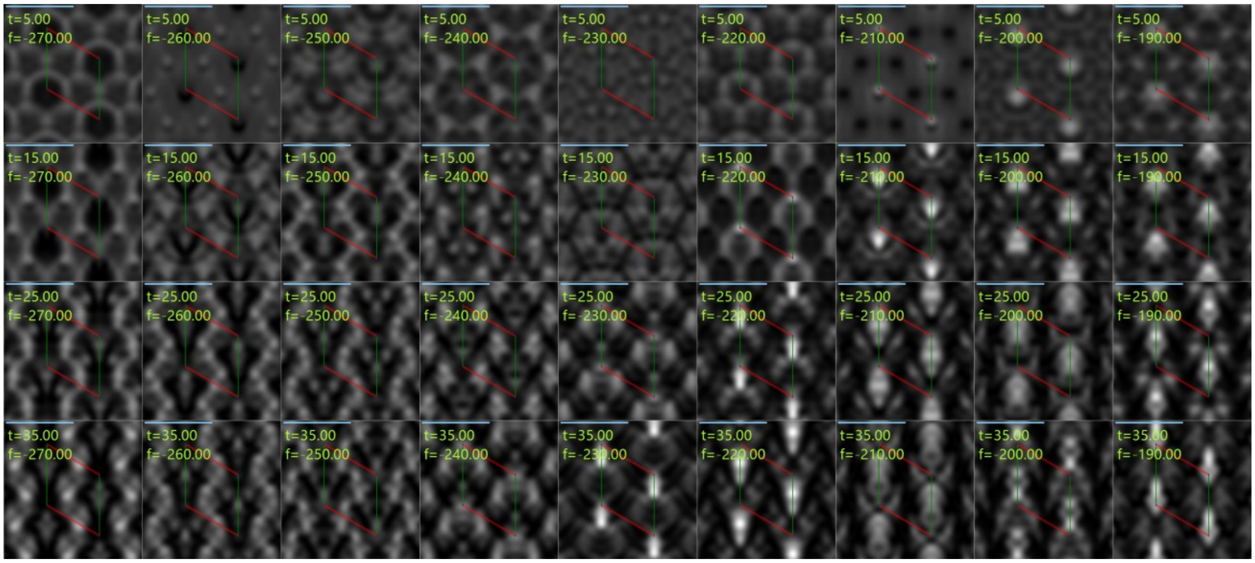

Supplementary Fig. 26. Kinematically simulated HRTEM image matrix with high defocus of ice I_h along the [0001] zone axis tilted by 0.8° towards the *a*$_2$-axis direction (vertical). Thickness (t) varies from 5 to 35 nm (by row). Defocus (f) varies from −270 to −190 nm (by column). Scale bars (cyan): 0.5 nm. A unit cell is outlined in each image.

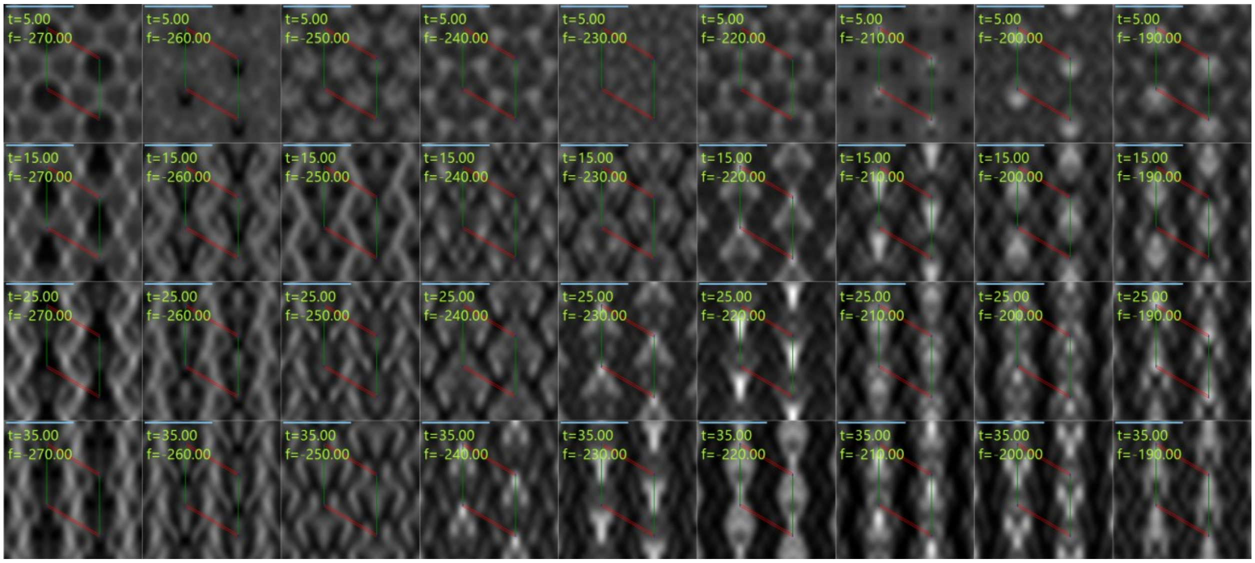

Supplementary Fig. 27. Kinematically simulated HRTEM image matrix with high defocus of ice I_h along the [0001] zone axis tilted by 1.2° towards the *a*$_2$-axis direction (vertical). Thickness (t) varies from 5 to 35 nm (by row). Defocus (f) varies from −270 to −190 nm (by column). Scale bars (cyan): 0.5 nm. A unit cell is outlined in each image.

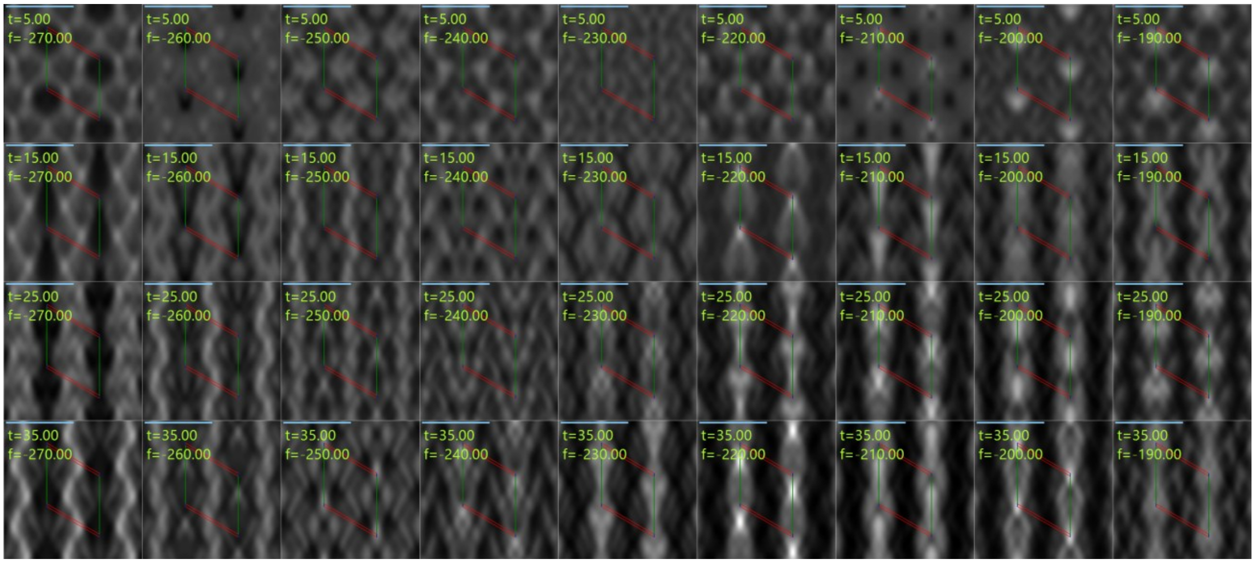

Supplementary Fig. 28. Kinematically simulated HRTEM image matrix with high defocus of ice I_h along the [0001] zone axis tilted by 1.6° towards the a_2-axis direction (vertical). Thickness (t) varies from 5 to 35 nm (by row). Defocus (f) varies from −270 to −190 nm (by column). Scale bars (cyan): 0.5 nm. A unit cell is outlined in each image.

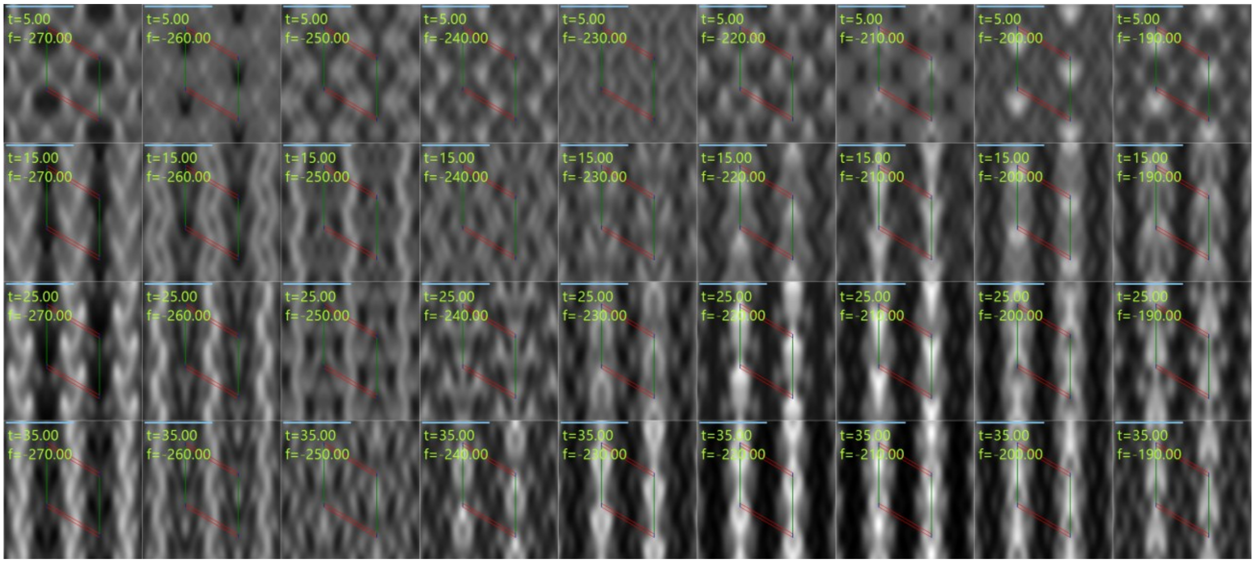

Supplementary Fig. 29. Kinematically simulated HRTEM image matrix with high defocus of ice I_h along the [0001] zone axis tilted by 2.0° towards the a_2-axis direction (vertical). Thickness (t) varies from 5 to 35 nm (by row). Defocus (f) varies from −270 to −190 nm (by column). Scale bars (cyan): 0.5 nm. A unit cell is outlined in each image.

Supplementary Note 1.8. _Multislice TEM simulation and Fourier transform_

Mathematically, the periodic lattice dot patterns in an HRTEM image convert to a dot array in reciprocal space. When there is a slight tilt from the crystal zone axis, an anisotropy in the lattice patterns emerges. As such, the strength of the periodic component in the tilt direction reduces relative to the orthogonal direction, which leads to an imbalance in the spot amplitude in the Fourier transform. This is the basis of the lattice amplitude mapping approach used in this study.

To further prove that this approach is appropriate for HRTEM images, we ran multislice TEM simulations for ice I_h crystals along the [0001] zone axis and those slightly tilted. Here, as the tilt angle increases, an increased anisotropy in the lattice dot patterns was observed, along with the reduced amplitude of the reflection spots in the vertical direction in the Fourier transform (Supplementary Fig. 30a–c). The amplitude ratio between the vertical and the two other $\{\bar{1}100\}$ spots show a gradual reduction as a function of increasing tilt angle (Supplementary Fig. 30d), which is consistent with the assumption that the amplitude balance between reflections represents the degree of tilt of the local crystal domain.

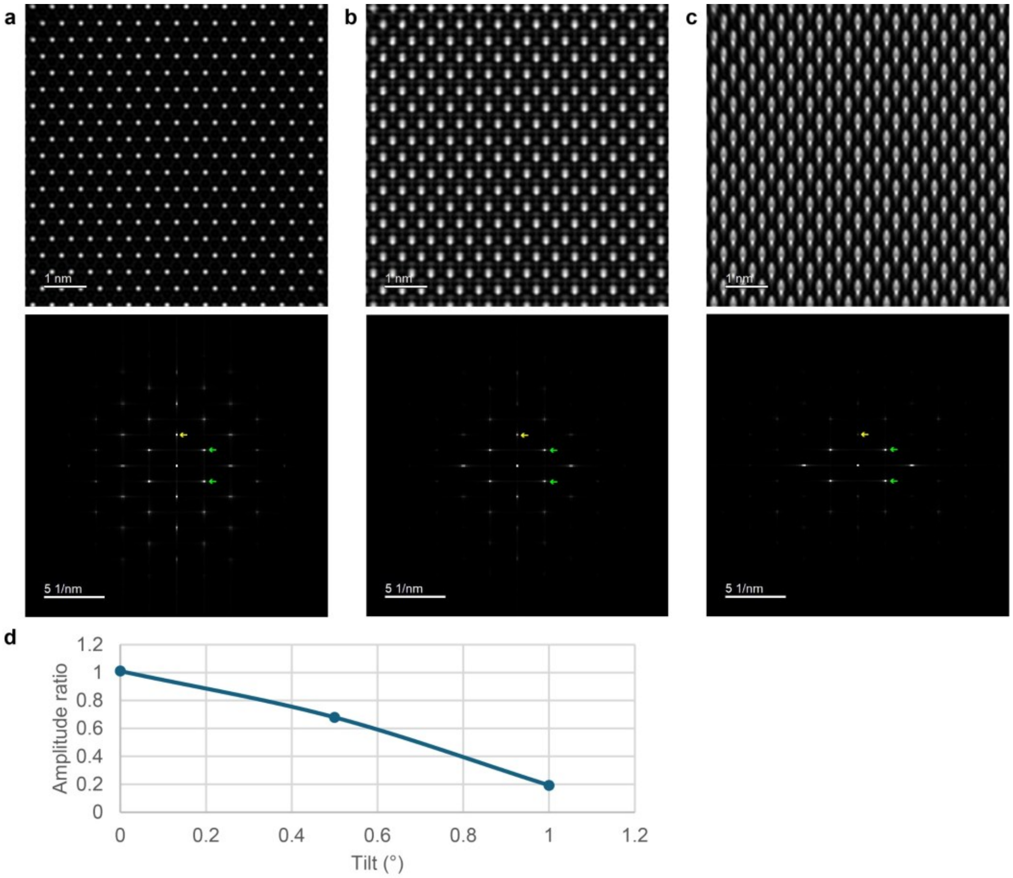

Supplementary Fig. 30. Multislice TEM simulation of tilted crystals. a–c, Multislice simulated TEM images (upper row) and corresponding Fourier transform (lower row) for an ice crystal viewed along the [0001] zone axis (**a**) and those tilted by 0.5° (**b**) or 1.0° (**c**). Crystal thickness = 25 nm. Defocus = −200 nm. **d,** The amplitude ratio of the vertical (yellow arrow in the Fourier transform images) and an average of the other two $\{\bar{1}100\}$ reflections (green arrows) as a function of the tilt angle.

Supplementary Note 1.9. *Bubble dynamics*

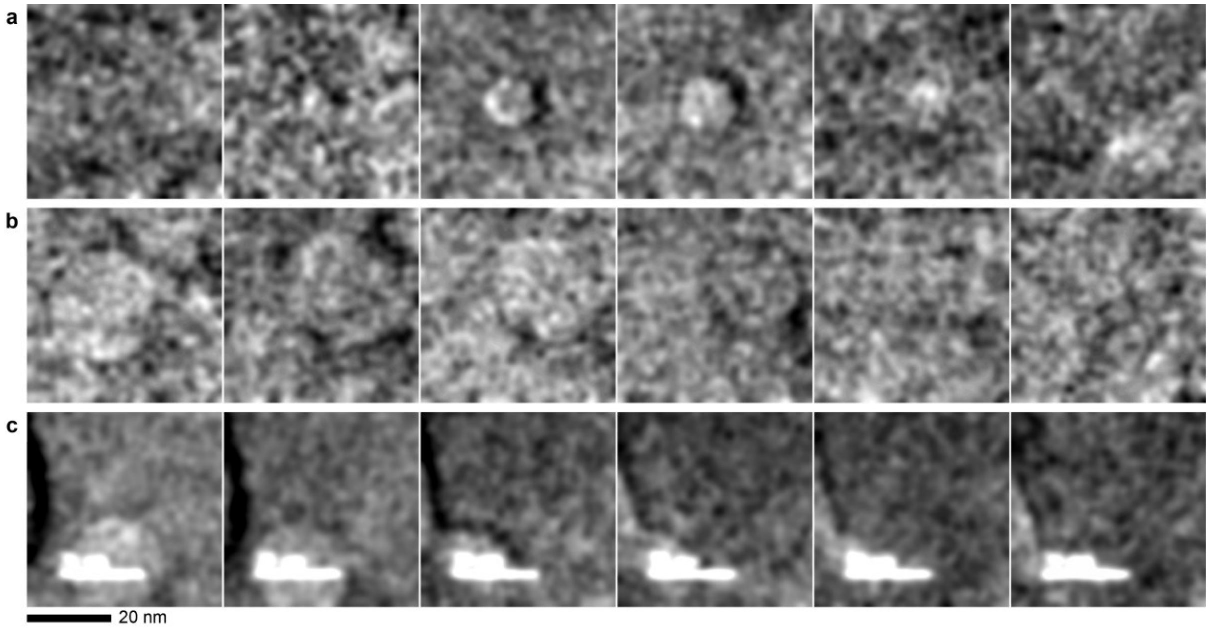

Supplementary Fig. 31. Lowpass-filtered HRTEM images for Fig. 5a to c. Cutoff frequency: 0.05 pixel^{-1} (0.465 nm^{-1}). White features in **c** are due to the embedded scale bars in the screen recording.

Supplementary Note 2. Additional Details for Molecular Dynamics Simulation

Supplementary Note 2.1. MD simulation details for tilt boundaries

To better understand the relationship between the tilt angle of the boundaries and their thermodynamic stability, we conducted MD simulations at temperatures emulating the experimental conditions. As depicted in Supplementary Fig. 32a, the simulation setup begins with a block of hexagonal ice with dimensions (l, h, w). We note that we have simulation setups for four different sizes with dimensions (105, 69, 21), (105, 69, 43), (105, 69, 65), (105, 69, 130), and finally a larger size (132, 69, 261). All dimensions are in Å. It is to be noted that for all the cases, the dimensions primarily vary along the height of the box. We denote these in terms of replication of the original supercell of the hexagonal ice we started with as (4 × 3 × 1), (4 × 3 × 2), (4 × 3 × 3), (4 × 3 × 6), (5 × 3 × 12). We tilted the half section of the initial block with the desired tilt angle. Depending on the size of the supercell, different concentrations of defects for the same tilt angle were introduced. We then used an annealing protocol to relax these boundaries and bring them to the desired temperatures, as depicted in Supplementary Fig. 32b. A typical annealed boundary is shown in Supplementary Fig. 32c. Different replication sizes are used to have a different degree of defect concentration in the starting configuration. As there is an inherent mismatch between tilted surfaces, this introduces defects.

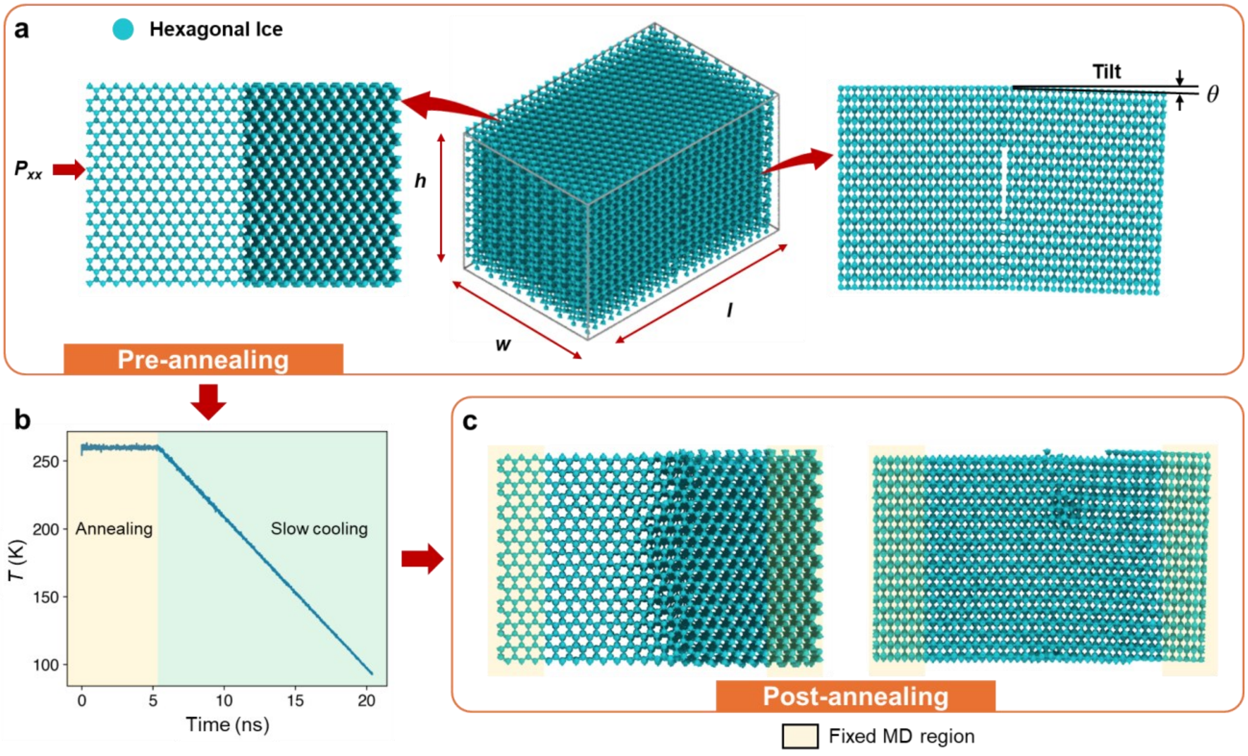

Supplementary Fig. 32. The overall MD simulation setup for grain boundaries. a, In the pre-annealing stage, a tilt angle boundary of the hexagonal ice is created by rotating the half section of the hexagonal ice block around the a_2 axis. **b,** Typical annealing profile used during the simulation. **c,** Top (left) and front (right) view of the final annealed configuration of a typical model.

Water molecules are represented as coarse-grained beads, where each molecule is simplified to one bead placed at the oxygen atom position with hydrogen atoms removed. Interactions between these water beads are described by a machine-learned Tersoff bond-order potential[7], which captures the properties of water phases with reasonable agreement with experiments. For a starting supercell configuration, we minimized it locally with the conjugate gradient algorithm[8] on the ML-BOP potential energy surface. The LAMMPS package[9] is used for all molecular dynamics simulations in the *NVE* ensemble. It is important to note that the particles at both ends of the simulation box were fixed during the time integration by setting their resultant forces to zero (Supplementary Fig. 32c). This was done to retain the initial tilt angle, mimicking two large grains with the desired tilt. Our focus is on the boundary region where two grains meet. Therefore, a Langevin thermostat was applied between the fixed sections. Additionally, we maintained a fixed pressure of 0 atm along P_{xx} (Supplementary Fig. 32a). The setup's post-minimized configuration was first annealed at 260 K to allow faster system relaxation and diffusion of defects at the boundary. This temperature was held for 5 ns, and then the system was gradually cooled to 93 K in the next 15 ns (Supplementary Fig. 32b). Finally, the pre- and post-annealing angles and energetics were computed.

To calculate the tilt angle between the minimized and post-annealing configurations, we began by selecting two spherical sections near the center from each side (tilted and non-tilted) of the simulation box for the initial, minimized, and relaxed configurations. These sections are essentially sets of Cartesian coordinates represented by ($N \times 3$) matrices. To determine the optimal rotation and translation between two sets of corresponding 3D point data, we sought the best transformation that aligns the points in matrix A to matrix B. This transformation is often referred to as the Euclidean or Rigid transform because it preserves shape and size. To find the angle, we need to determine the rotation matrix R. As we have only rotation in one direction, we can calculate the tilt from the rotation matrix afterward. Singular value decomposition (SVD) can be used to find the subsequent rotation matrix[10]. We obtained a covariance matrix H such that:

$$H = \; A - \mathrm{Centroid}_A \;\; B - \mathrm{Centroid}_B \;^{\intercal}$$
$$[U, S, V] = \mathrm{SVD}\; H$$
$$R = VU^{\intercal}$$

Supplementary Eq. (2)

Supplementary Fig. 33 shows the variation of potential energy for different sizes and tilt angles throughout the simulation duration. It can be observed that during the annealing time of the first 5 ns, the potential energy remains constant and decreases subsequently as the system cools down. One noticeable aspect is that at lower tilt angles (Supplementary Fig. 33a and b), the energies of all sizes almost overlap. This is because the concentration of defects at lower tilt angles, irrespective of the size of the supercell, is very low. As the tilt angle increases, so does the dislocation defect concentration with the increase in the supercell size (Supplementary Fig. 33c to f). This causes varying energies for different sizes as the simulation progresses. We note that for almost all cases, the largest supercell ($5 \times 4 \times 12$) eventually reaches an energy lower than all of its peers. This reveals that low-angle boundaries can be stabilized by dislocations with very low energy penalties. Rather than increasing the boundary energy, higher tilt angles lead to the creation of new dislocations, resulting in a reduction of the system's energy. This allows for the formation of diverse interfaces without incurring significant penalties.

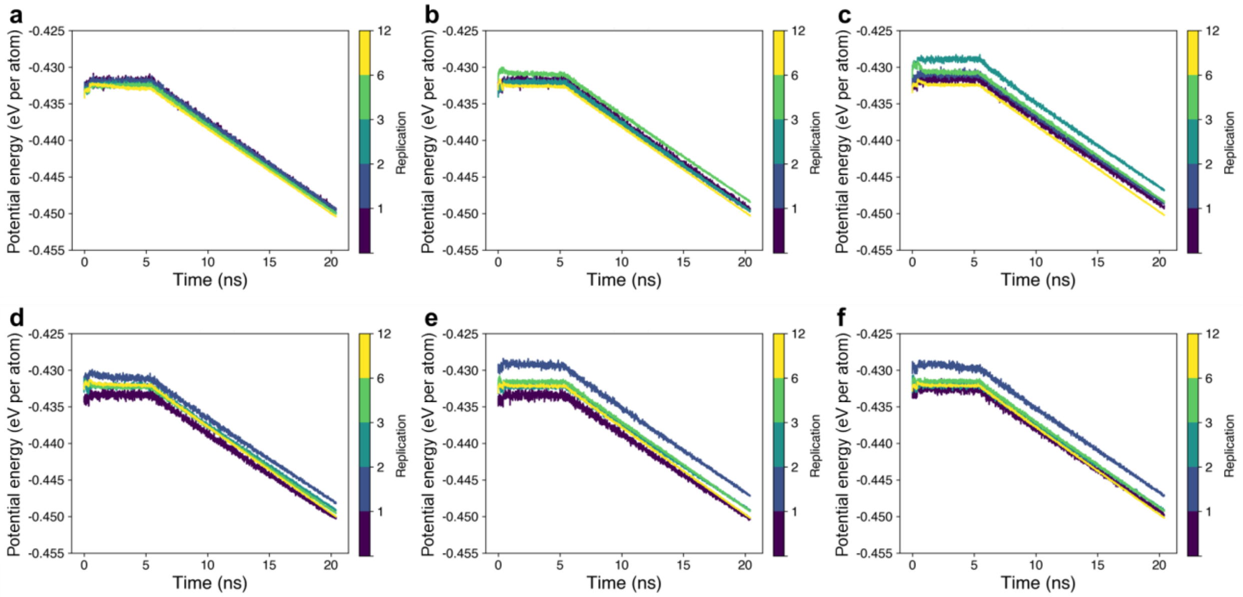

Supplementary Fig. 33. Variation of the potential energy with time for different sizes of configurations and tilt angles. Plotted are the potential energy per molecule against the simulation time for initial tilt angles of 0.2° (**a**), 0.6° (**b**), 1.2° (**c**), 1.6° (**d**), 1.8° (**e**), and 2.0° (**f**), respectively.

Supplementary Table 1. Equilibrated tilt angle and mean cohesive energy of the low-angle grain boundary with *h* = 1 from the MD simulation at 93 K after annealing.

Initial tilt (°)	Equilibrated tilt (°)	Mean cohesive energy (eV per atom)
0.0	0.66305	−0.45054
0.2	0.48696	−0.44938
0.4	0.12359	−0.44939
0.6	0.31435	−0.44940
0.8	0.03098	−0.44919
1.0	0.06518	−0.44929
1.2	0.34492	−0.44917
1.4	0.13814	−0.44833
1.6	0.07485	−0.45018
1.8	0.22442	−0.45034
2.0	0.58875	−0.44976

Supplementary Table 2. Equilibrated tilt angle and mean cohesive energy of the low-angle grain boundary with *h* = 2 from the MD simulation at 93 K after annealing.

Initial tilt (°)	Equilibrated tilt (°)	Mean cohesive energy (eV per atom)
0.0	0.1754	−0.45058
0.2	0.11199	−0.44949
0.4	0.01689	−0.44979
0.6	0.39062	−0.44970
0.8	0.18748	−0.44959

1.0	0.50077	−0.44990
1.2	0.4724	−0.44861
1.4	0.26985	−0.44863
1.6	0.3494	−0.44812
1.8	0.36574	−0.44715
2.0	3.11576	−0.44710

Supplementary Table 3. Equilibrated tilt angle and mean cohesive energy of the low-angle grain boundary with *h* = 3 from the MD simulation at 93 K after annealing.

Initial tilt (°)	Equilibrated tilt (°)	Mean cohesive energy (eV per atom)
0.0	0.13170	−0.45052
0.2	0.13512	−0.44989
0.4	0.17156	−0.44985
0.6	0.32064	−0.44963
0.8	0.46252	−0.44925
1.0	0.69297	−0.44750
1.2	0.83801	−0.44683
1.4	2.10579	−0.44770
1.6	1.98957	−0.44914
1.8	1.91082	−0.44920
2.0	2.10446	−0.44913

Supplementary Table 4. Equilibrated tilt angle and mean cohesive energy of the low-angle grain boundary with *h* = 4 from the MD simulation at 93 K after annealing.

Initial tilt (°)	Equilibrated tilt (°)	Mean cohesive energy (eV per atom)
0.0	0.02520	−0.45052
0.2	0.09408	−0.45003
0.4	0.33899	−0.44908
0.6	0.81075	−0.44841
0.8	0.85889	−0.44882
1.0	0.96212	−0.44890
1.2	1.47930	−0.44839
1.4	1.87475	−0.44880
1.6	1.69638	−0.44951
1.8	1.93293	−0.44924
2.0	2.28449	−0.44921

Supplementary Table 5. Equilibrated tilt angle and mean cohesive energy of the low-angle grain boundary with *h* = 5 from the MD simulation at 93 K after annealing.

Initial tilt (°)	Equilibrated tilt (°)	Mean cohesive energy (eV per atom)
0.0	0.01754	−0.45151
0.2	0.19995	−0.45041

0.4	0.61409	−0.45023
0.6	0.99410	−0.45027
0.8	0.68264	−0.45034
1.0	1.03049	−0.45048
1.2	0.89977	−0.45023
1.4	1.61049	−0.45021
1.6	1.60362	−0.45000
1.8	1.68874	−0.45018
2.0	1.85593	−0.45017

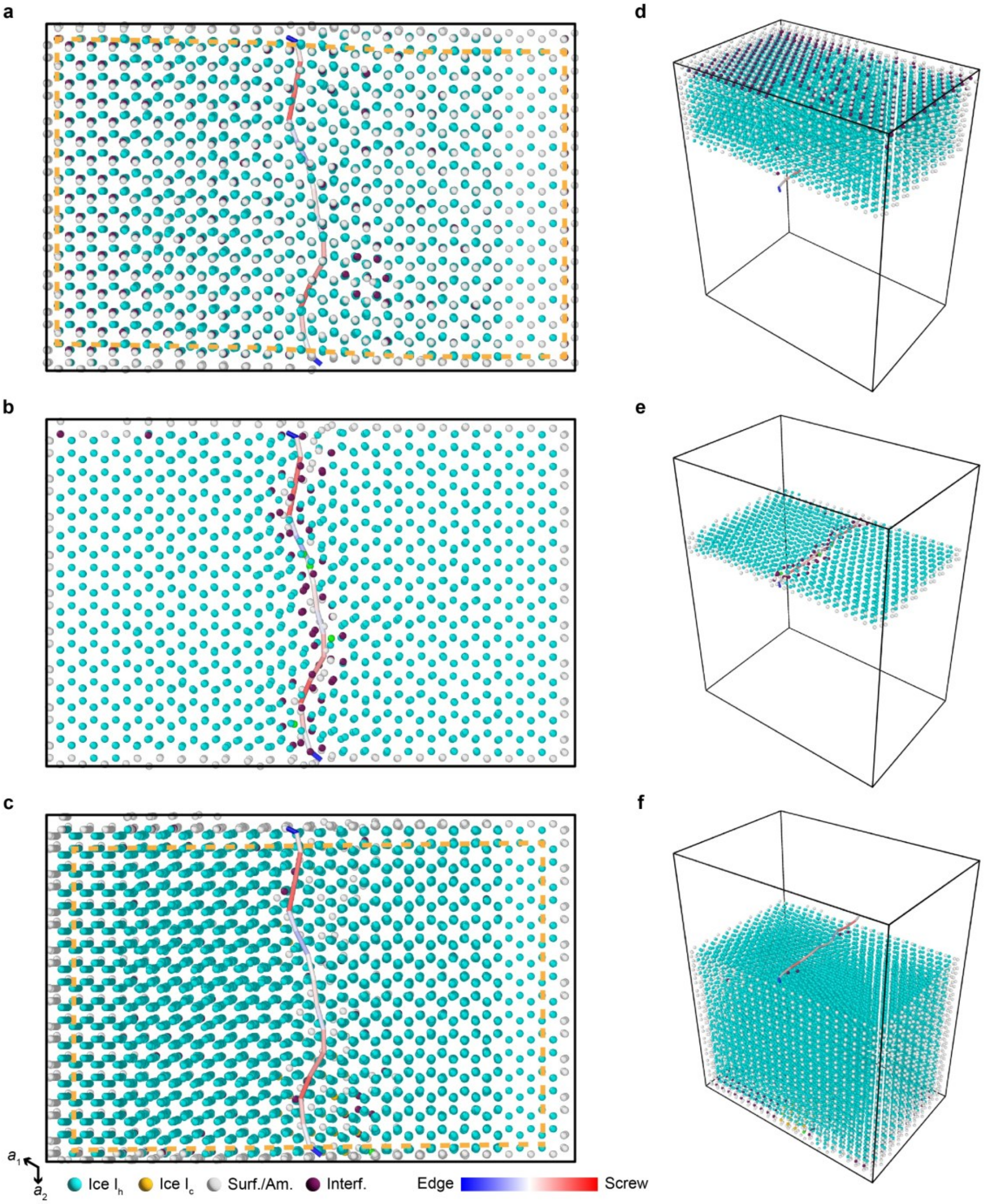

Supplementary Fig. 34. Structural analysis of the mixed screw and edge dislocation from the MD simulation (*h* = 6, initial tilt angle = 1.0°, final tilt angle = 0.96°). a–c, Top view of the cross-sectional models of the upper domain (**a**), interfacial layers (**b**), and the lower domain (**c**). Orange dashes outline the lattices and show the half-plane mismatch between the upper and the lower domains. **d–f,** Corresponding perspective models. Beads: water molecules in ice, surface/amorphous (surf./am.), interfacial (interf.), or hydrate-like local configurations (other colors). The local character of the dislocation is quantified by a color scale (blue-white-red).

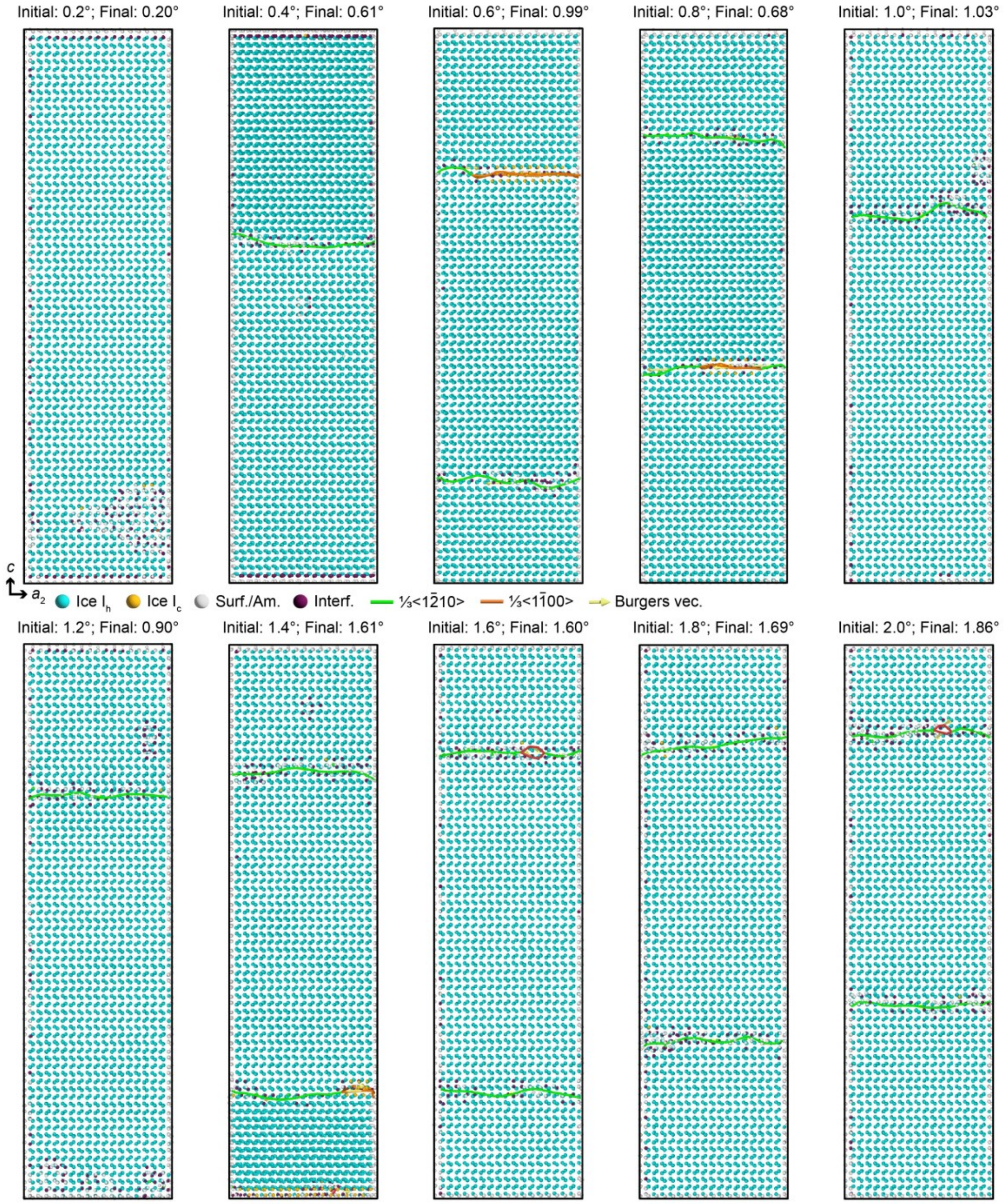

Supplementary Fig. 35. Cross-sectional models of the tilted ice (*h* = 12) with various tilt angles. Beads: water molecules in ice, surface/amorphous (surf./am.), interfacial (interf.), or hydrate-like local configurations (other colors). Red lines represent dislocations of other types.

Supplementary Note 2.2. *MD simulation details for bubbles/cavities*

We conducted molecular dynamics (MD) simulations of nanobubbles using LAMMPS in a 20 nm × 20 nm × 20 nm orthogonal simulation box with periodic boundary conditions. To create the initial structure of a nanobubble, we start with a hexagonal ice crystal lattice with its $(1\bar{1}00)$, $(11\bar{2}0)$, and (0001) planes, i.e., 1st prism, 2nd prism, and basal planes, normal to the *x*-, *y*-, and *z*-axes of the simulation box. Water molecules within a 6 nm distance from the center of the simulation box are removed to form the nanobubble. Subsequently, the initial nanobubble structure undergoes energy minimization and equilibration for 50 ps in an isothermal-isobaric ensemble at a temperature of 260 K and pressure of 1 bar. In this equilibration step and all subsequent steps, the simulation timestep is set to 5 fs, and the Nose-Hoover thermostat and barostat damping time constants are set to 0.1 ps and 1.0 ps, respectively. After the initial equilibration, a series of fixed-volume (canonical ensemble) equilibrations are applied only to the water beads within a 9 nm distance from the center of the simulation box. This process involves heating for 0.5 ns to raise the temperature from 260 K to 270 K, followed by a 50 ps structural relaxation at 370 K, a 10 ns quenching step to decrease the temperature from 370 K back to 260 K, and finally, a 2 ns structural relaxation at 260 K to obtain the final structure of the static nanobubble.

Supplementary Note 2.3. *Facet recognition for bubbles*

Water molecules in the first crystalline layer on the surface of the simulated static nanobubble are labeled as one of three hexagonal ice surface types (basal, primary prism, and secondary prism) based on a score for each water molecule. This score determines if its neighbors within a 6-Å cutoff radius lie on a plane parallel to any of the ice surface planes. To determine the scores, we first obtain unit normal vectors of the families of Miller-Bravais planes $\{hkil\}$ relevant to the hexagonal ice surfaces. Since the *x*-, *y*-, and *z*-axes of our simulation box are normal to the $(1\bar{1}00)$, $(11\bar{2}0)$, and (0001) planes of the simulated hexagonal ice crystal lattice, the unique unit vectors, n̂, for non-parallel planes are [0, 0, 1] for the (0001) basal planes, [1/2, $\sqrt{3}$/2, 0], [1, 0, 0], [1/2, -$\sqrt{3}$/2, 0] for the $(10\bar{1}0)$, $(1\bar{1}00)$, $(0\bar{1}10)$ 1st prism planes, and [0, 1, 0], [$\sqrt{3}$/2, 1/2, 0], [$\sqrt{3}$/2, -1/2, 0] for the $(11\bar{2}0)$, $(2\bar{1}\bar{1}0)$, $(1\bar{2}10)$ 2nd prism planes. For every water molecule, we compute all vectors between point ***A*** (the molecule itself) and point ***B*** (its neighbors) and calculate the cosine between these vectors and the surface normal vector through the dot product,

$$\text{cosine}_{hkil} = \overline{\boldsymbol{AB}}_{\text{unit}} \cdot \hat{\text{n}}.$$

Supplementary Eq. (3)

For a given molecule, if it were to lie on a given plane, the unit vector $\overline{\boldsymbol{AB}}_{\text{unit}}$ for all the neighboring molecules should be perpendicular to the surface normal vector, making the cosine close to 0. Additionally, to account for thermal noise, a lower standard deviation among their cosine distances means that they will be parallel to the surface, and this facet is more prominent. Thus, the final score is determined as follows:

$$\text{score}_{hkil} = \text{SD}\left(\text{cosine}_{hkil}^{p} \in p = 1, \ldots, N\right) + \text{mean}\left(\text{cosine}_{hkil}^{p} \in p = 1, \ldots, N\right).$$

Supplementary Eq. (4)

We repeat the same process for all surface normal vectors and assign the surface type labels based on two criteria. If the overall score is > 0.6, do not assign the molecule to any surface; otherwise, assign the molecule to the surface having the lowest score associated with it.

Supplementary Note 2.4. Surface energy calculation

The individual slabs with the c lattice vector oriented normal to the target plane were constructed using Python libraries pymatgen[11] and spglib[12] from a unit cell of hexagonal ice (I_h). The energies of the unit cell and the slab were computed using the LAMMPS, employing the ML-BOP model[7]. The surface energy for a facet with Miller-Bravais indices $\{hkil\}$ is:

$$\mathrm{SE}_{hkil} = \frac{E_{\mathrm{slab}} - nE_{\mathrm{pa,unit}}}{2A},$$

Supplementary Eq. (5)

where E_{slab} is the total cohesive energy of the slab, n is the number of molecules in the slab, $E_{\mathrm{pa,unit}}$ is the energy per molecule of the unit cell of hexagonal ice, and A is the exposed surface area of the slab.

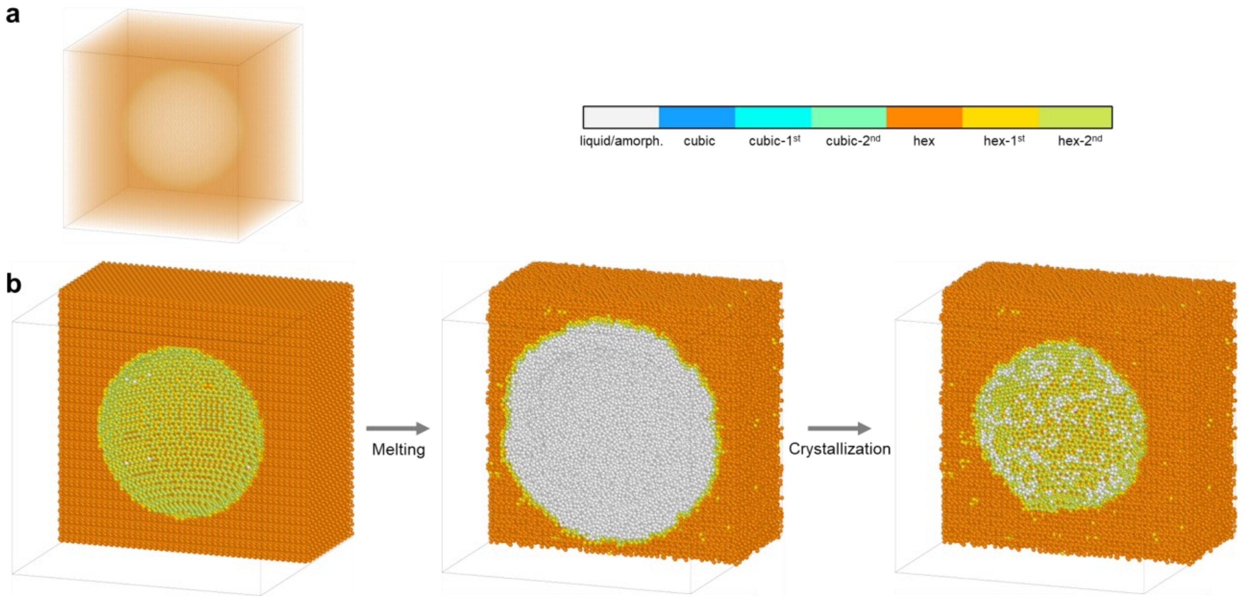

Supplementary Fig. 36. MD modeling of a nanobubble in ice I_h. a, Illustration of the initial setup with a spherical cavity inside an ice crystal. **b,** Cross-sectional view of the model in the MD simulation for obtaining an equilibrated structure. Color coding represents the local environment of the molecules. Labels "-1st" and "-2nd" refer to molecules that are the first or second nearest neighbors of another molecule that has been identified as a cubic or hexagonal lattice site, but at least one of the nearest neighbors is not at a lattice site.

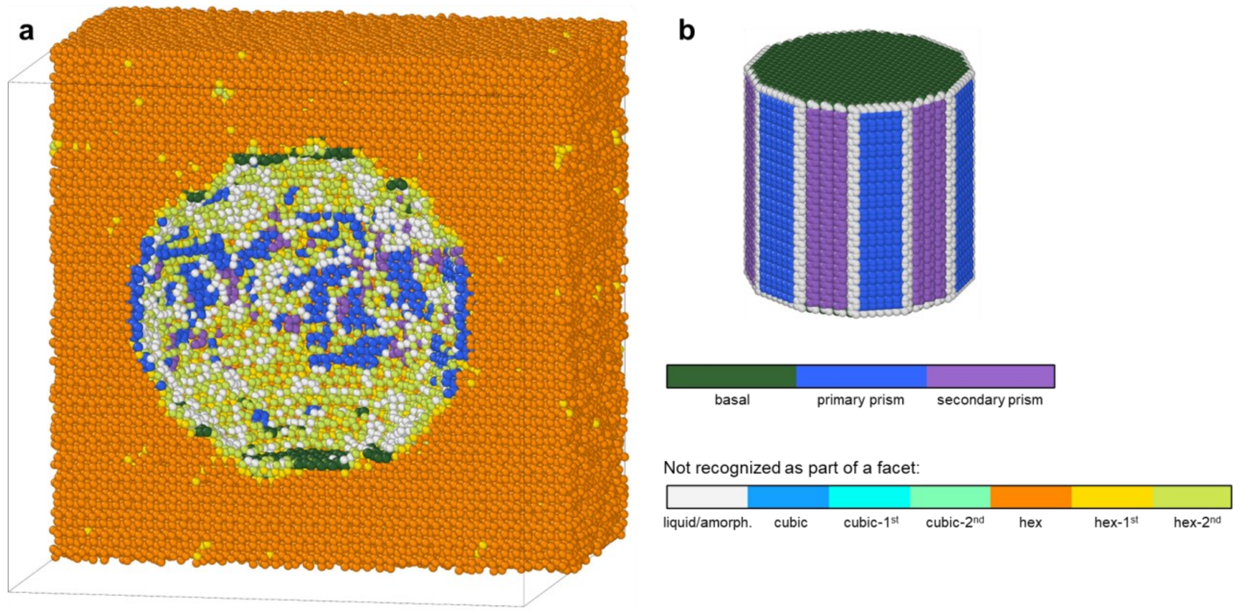

Supplementary Fig. 37. Facet recognition of MD-simulated nanobubble. a, Cross-sectional model. **b,** Color-coding for facets illustrated in a prismatic crystal.

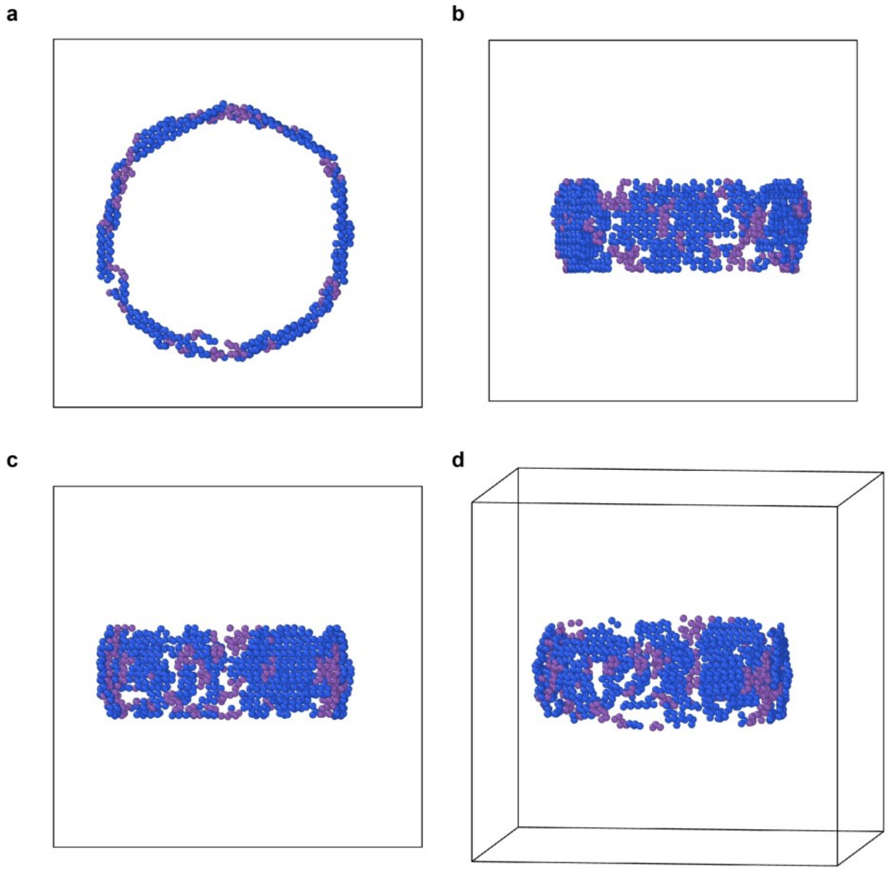

Supplementary Fig. 38. Cross-section (5-nm in the center) of MD-simulated bubble surface colored by recognized facets in different views. a, Top. **b,** Left. **c,** Front. **d,** 3D. Vertical direction: *c*-axis.

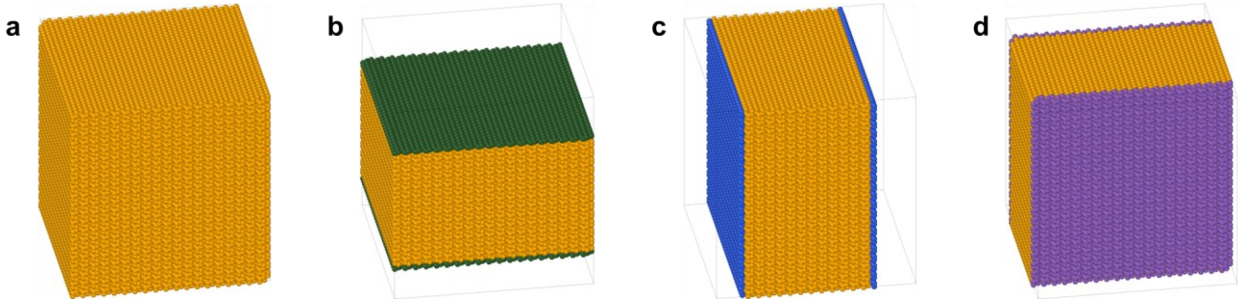

Supplementary Fig. 39. MD models used for evaluating surface energies of different facets. a, Bulk crystal. **b,** Basal planes. **c,** Primary prism planes. **d,** Secondary prism planes.

Supplementary Table 6. Summary of surface energy calculations from MD. PE: cohesive energy.

Surface type	# of molecules	PE (eV)	Surface area (Å^2)	$PE_{surface}$ (meV Å^{-2})
Bulk reference	105335	−48826.249	0	0
Basal	67715	−31107.526	43340.01	6.475
Primary prism	72071	−33100.841	45162.60	6.785
Secondary prism	71819	−32938.276	45037.91	7.820

Supplementary Note 3. Additional Details for Theoretical Calculations

Supplementary Note 3.1. *Elastic strain*

According to the continuum theories of elasticity[13], for a spherical cavity (radius = R) inside an isotropic material with an internal pressure of the cavity p_1 and external pressure p_2, components of the strain tensor in spherical polar coordinates take the form of:

$$\varepsilon_{rr} = a - \frac{2b}{r^3}, \varepsilon_{\theta\theta} = \varepsilon_{\phi\phi} = a + \frac{b}{r^3}.$$

Supplementary Eq. (6)

Accordingly, the radial stress is:

$$\sigma_{rr} = \frac{E}{1-2\nu}a - \frac{2E}{1+\nu}\frac{b}{r^3},$$

Supplementary Eq. (7)

where E is the Young's modulus and ν is the Poisson's ratio. Given stress boundary conditions $\sigma_{rr}|_{r=R} = -p_1$ and $\sigma_{rr}|_{r\to+\infty} = -p_2$, a and b are determined by:

$$a = \frac{p_2}{E}\ 2\nu - 1\ , b = \frac{p_1 - p_2}{2E}\ 1 + \nu\ R^2.$$

Supplementary Eq. (8)

Now we consider a Laplacian pressure caused by the spherical cavity surface:

$$\Delta P = p_1 - p_2 = \frac{2\gamma}{R},$$

Supplementary Eq. (9)

and assume atmospheric pressure outside the material $p_2 = p^{\varnothing}$. Plugging these values into Supplementary Eq. (6) gives:

$$\varepsilon_{rr} = \frac{p^{\varnothing}}{E}\ 2\nu - 1\ + \frac{2\gamma R^2}{Er^3}\ 1 + \nu\ ,$$
$$\varepsilon_{\theta\theta} = \varepsilon_{\phi\phi} = \frac{p^{\varnothing}}{E}\ 2\nu - 1\ - \frac{\gamma R^2}{Er^3}\ 1 + \nu\ .$$

Supplementary Eq. (10)

Strain components on the surface can be obtained by evaluating it at $r = R$:

$$\varepsilon_{rr}|_{r=R} = \frac{p^{\varnothing}}{E}\ 2\nu - 1\ + \frac{2\gamma}{ER}\ 1 + \nu\ ,$$
$$\varepsilon_{\theta\theta}|_{r=R} = \varepsilon_{\phi\phi}|_{r=R} = \frac{p^{\varnothing}}{E}\ 2\nu - 1\ - \frac{\gamma}{ER}\ 1 + \nu\ .$$

Supplementary Eq. (11)

For ice, we take a slightly overestimated surface energy[14,15] $\gamma = 200$ mJ m^{-2}, Young's Modulus[16] $E =$ 11 GPa at −180 °C, and Poisson's ratio[17] $\nu = 0.33$. The radial and tangential strains in the material as a function of the distance to the cavity surface (Δr) are plotted in Supplementary Fig. 40. The strain components on the cavity surface are calculated by Supplementary Eq. (11) and given in Supplementary Table 7.

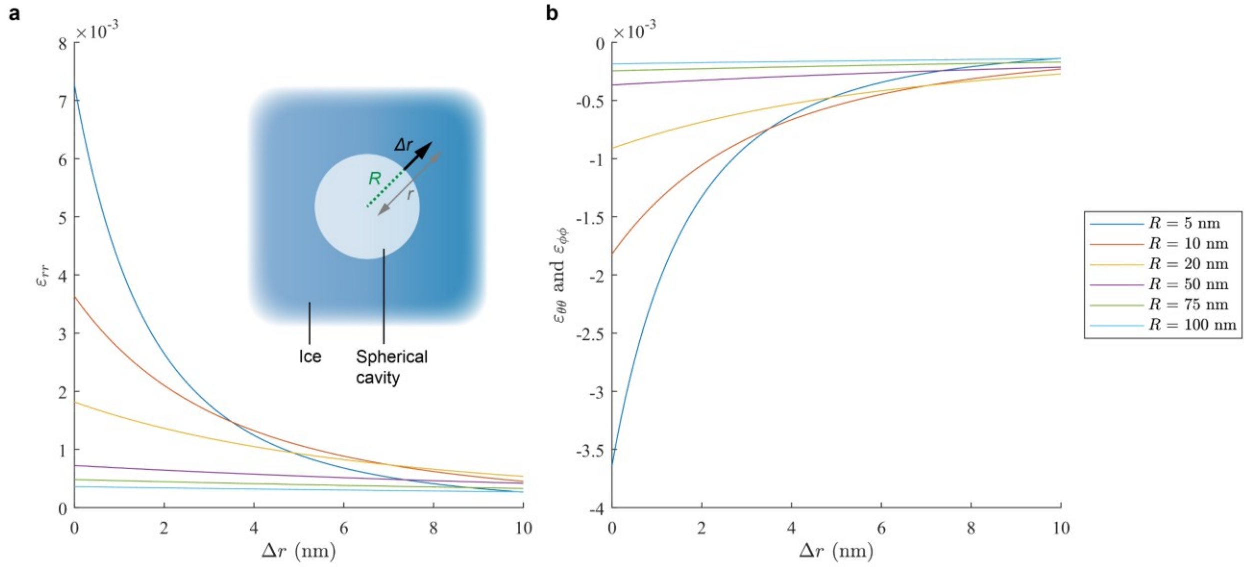

Supplementary Fig. 40. Elastic strain field around a spherical cavity in ice based on continuum theories. a, Radial elastic strain. **b,** Tangential elastic strain. Δr is the distance to the cavity surface, i.e., $r = R + \Delta r$.

Supplementary Table 7. Elastic strain components on the cavity surface in ice based on continuum theories.

R (nm)	$\boldsymbol{\varepsilon_{rr}\vert_{r=R}}$	$\boldsymbol{\varepsilon_{\theta\theta}\vert_{r=R}}$ and $\boldsymbol{\varepsilon_{\phi\phi}\vert_{r=R}}$
5	0.007270	−0.00364
10	0.003633	−0.00182
20	0.001815	−0.00091
50	0.000724	−0.00037
75	0.000482	−0.00025
100	0.000361	−0.00018

Supplementary Note 3.2. Radiolysis calculation

The temperature-dependent kinetic model assumes that the reaction rates k follow an Arrhenius behavior given by an activation energy E_A and an Arrhenius factor A (R is the gas constant and T the temperature)[18]:

$$k = A\mathrm{e}^{\frac{E_A}{\mathrm{R}T}}$$

Supplementary Eq. (12)

The included reactions and corresponding values for A and E_A are listed in Supplementary Table 8. These parameters were acquired for temperatures between 25 and 100 °C. Consequently, applying these rate constants to lower temperatures is an extrapolation of Supplementary Eq. (12).

Supplementary Table 8. Chemical reactions used in the kinetic model[18]. In the Arrhenius factor A, n describes the reaction order.

	Reaction	A (mol^{-n+1} L$^{(n-1)}$ s^{-1})	E_A (kJ mol^{-1})
1	$H^+ + OH^- \longrightarrow H_2O$	1.88×10^{13}	12.62
2	$H_2O \longrightarrow H^+ + OH^-$	1.70×10^{6}	62.37
3	$H_2O_2 \longrightarrow H^+ + HO_2^-$	4.12×10^{6}	43.77
4	$H^+ + HO_2^- \longrightarrow H_2O_2$	5.59×10^{12}	11.73
5	$H_2O_2 + OH^- \longrightarrow HO_2^- + H_2O$	3.66×10^{12}	13.98
6	$HO_2^- + H_2O \longrightarrow H_2O_2 + OH^-$	4.54×10^{11}	31.74
7	$e_h^- + H_2O \longrightarrow H + OH^-$	5.58×10^{6}	31.73
8	$H + OH^- \longrightarrow e_h^- + H_2O$	8.52×10^{13}	37.36
9	$H \longrightarrow H^+ + e_h^-$	2.84×10^{12}	66.66
10	$H^+ + e_h^- \longrightarrow H$	1.98×10^{12}	11.17
11	$HO_2 \longrightarrow O_2^- + H^+$	2.63×10^{8}	14.58
12	$O_2^- + H^+ \longrightarrow HO_2$	5.59×10^{12}	11.73
13	$HO_2 + OH^- \longrightarrow O_2^- + H_2O$	7.13×10^{9}	60.93
14	$O_2^- + H_2O \longrightarrow HO_2 + OH^-$	3.66×10^{12}	13.98
15	$e_h^- + OH \longrightarrow OH^-$	2.64×10^{12}	10.65
16	$e_h^- + H_2O_2 \longrightarrow OH + OH^-$	7.75×10^{12}	15.72
17	$e_h^- + H_2O + O_2^- \longrightarrow HO_2^- + OH^-$	4.43×10^{10}	12.98
18	$e_h^- + HO_2 \longrightarrow HO_2^-$	2.45×10^{12}	12.98
19	$e_h^- + O_2 \longrightarrow O_2^-$	2.53×10^{12}	11.66
20	$2\ e_h^- + 2\ H_2O \longrightarrow H_2 + 2\ OH^-$	1.01×10^{10}	20.74
21	$e_h^- + H + H_2O \longrightarrow H_2 + OH^-$	2.06×10^{11}	14.93
22	$H + H_2O \longrightarrow H_2 + OH$	7.39×10^{12}	98.24
23	$2\ H \longrightarrow H_2$	2.69×10^{12}	15.51
24	$H + OH \longrightarrow H_2O$	4.19×10^{11}	9.03
25	$H + H_2O_2 \longrightarrow OH + H_2O$	1.76×10^{11}	21.01
26	$H + O_2 \longrightarrow HO_2$	9.01×10^{11}	10.52
27	$H + HO_2 \longrightarrow H_2O_2$	5.05×10^{12}	15.09
28	$H + O_2^- \longrightarrow HO_2^-$	5.05×10^{12}	15.09
29	$2\ OH \longrightarrow H_2O_2$	9.78×10^{10}	7.48

30	$OH + HO_2 \longrightarrow O_2 + H_2O$	1.31×10^{11}	6.68
31	$OH + O_2^- \longrightarrow OH^- + O_2$	8.75×10^{11}	10.84
32	$H_2 + OH \longrightarrow H + H_2O$	6.55×10^{10}	18.45
33	$OH + H_2O_2 \longrightarrow HO_2 + H_2O$	7.72×10^{9}	13.82
34	$OH + HO_2^- \longrightarrow HO_2 + OH^-$	1.00×10^{12}	11.92
35	$HO_2 + O_2^- \longrightarrow HO_2^- + O_2$	2.62×10^{9}	8.09
36	$2\ HO_2 \longrightarrow O_2 + H_2O_2$	2.77×10^{9}	20.07

As the inelastic scattering of high-energy electrons in amorphous ice is close to water[19,20], room-temperature *G*-values are chosen for the simulation (Supplementary Table 9). We note, however, that *G*-values are known to change as a function of temperature, which has been approximated linearly[10]. Yet, linear extrapolation was ruled out for cryogenic temperatures, as this would quickly yield physically unreasonable results.

Supplementary Table 9. *G*-values used in this work[21].

Reactant	e_h^-	H^+	OH^-	H	OH	HO_2	H_2	H_2O_2	H_2O
G_i (10^{-2} molecules eV^{-1})	2.60	3.10	0.50	0.66	2.70	0.02	0.45	0.70	−4.64

All simulations based on Eq. (1) (Methods section) were conducted for 1000 s, sufficient to ensure steady state formation for all reactants. The evolution of chemical species under the experimental conditions of in situ bubble generation is given in Fig. 5f. The steady state at different temperatures at an electron flux of 25 e $Å^{-2}$ s^{-1} is given in Supplementary Fig. 41.

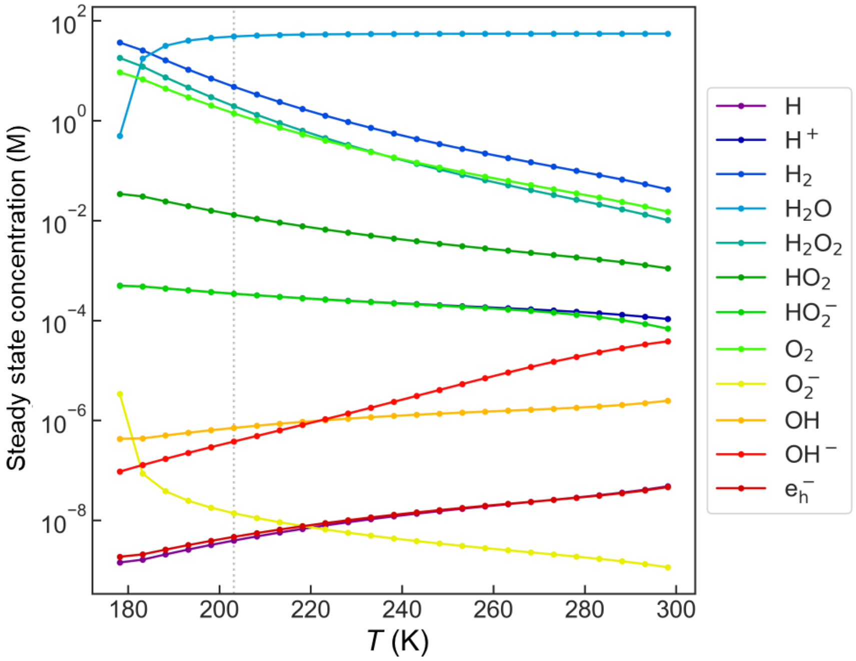

Supplementary Fig. 41. Radiolysis steady states as a function of temperature (*T*). The dotted vertical line denotes the temperature of in situ bubble generation in this work.

The steady-state concentrations of the stable main products of water radiolysis (H_2, O_2, H_2O_2) increase with cooling (Supplementary Fig. 42). Similarly, the ratio between H_2 and O_2, presumably the dominant gas species, also increases with temperature reduction. Consequently, mass balance eventually causes substantial water depletion. Here, the computation of the kinetic model fails because the physicochemical principles behind the model assume the primary interaction of electrons with water. If water depletes, the used *G*-values break down. In the present case, steady states were successfully simulated down to about 180 K. We note that this does not necessarily mean that at lower *T*, no steady state is expected; however, a model considering irradiation of H_2O alone is not feasible to describe the system accurately.

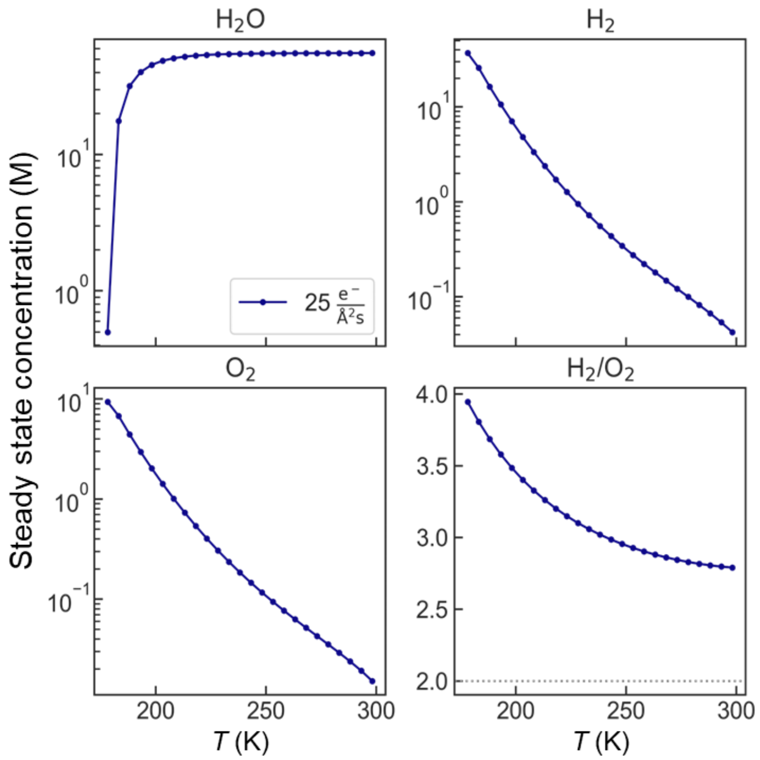

Supplementary Fig. 42. Steady-state concentrations of water and main gas components from radiolysis calculations as a function of temperature (*T*). The molar ratio between H_2 and O_2 increases with temperature reduction.

Noteworthy is the increase in the steady-state concentration of O_2 with reduced temperature, which is in good agreement with EELS measurements in cryo-TEM[22]. This indicates that the model is feasible for drawing qualitative conclusions despite the discussed approximations and limitations.

Supplementary References

1 Morgan, D. J. Comments on the XPS Analysis of Carbon Materials. *C-J. Carbon Res.* **7**, 51 (2021).

2 Stalder, A. F. *et al.* Low-bond axisymmetric drop shape analysis for surface tension and contact angle measurements of sessile drops. *Colloids Surf. A* **364**, 72-81 (2010).

3 Prydatko, A. V., Belyaeva, L. A., Jiang, L., Lima, L. M. C. & Schneider, G. F. Contact angle measurement of free-standing square-millimeter single-layer graphene. *Nat. Commun.* **9**, 4185 (2018).

4 Yonekura, K., Braunfeld, M. B., Maki-Yonekura, S. & Agard, D. A. Electron energy filtering significantly improves amplitude contrast of frozen-hydrated protein at 300kV. *J. Struct. Biol.* **156**, 524-536 (2006).

5 Fraundorf, P., Qin, W., Moeck, P. & Mandell, E. Making sense of nanocrystal lattice fringes. *J. Appl. Phys.* **98**, 114308 (2005).

6 Mallick, S. P., Carragher, B., Potter, C. S. & Kriegman, D. J. ACE: Automated CTF Estimation. *Ultramicroscopy* **104**, 8-29 (2005).

7 Chan, H. *et al.* Machine learning coarse grained models for water. *Nat. Commun.* **10**, 379 (2019).

8 Shewchuk, J. R. An introduction to the conjugate gradient method without the agonizing pain. (Carnegie Mellon University, 1994).

9 Thompson, A. P. *et al.* LAMMPS-a flexible simulation tool for particle-based materials modeling at the atomic, meso, and continuum scales. *Comput. Phys. Commun.* **271**, 108171 (2022).

10 Arun, K. S., Huang, T. S. & Blostein, S. D. Least-squares fitting of two 3-D point sets. *IEEE Trans. Pattern Anal. Mach. Intell.* **PAMI-9**, 698-700 (1987).

11 Ong, S. P. *et al.* Python Materials Genomics (pymatgen): A robust, open-source python library for materials analysis. *Comput. Mater. Sci.* **68**, 314-319 (2013).

12 Togo, A., Shinohara, K. & Tanaka, I. Spglib: a software library for crystal symmetry search. arXiv:1808.01590 (2018). <https://arxiv.org/abs/1808.01590>.

13 Landau, L. D., Lifshitz, E. M., Kosevich, A. M. & Pitaevskii, L. P. in *Theory of Elasticity* Vol. 7 *Course of Theoretical Physics* Ch. 1, 1-37 (Butterworth-Heinemann, 1986).

14 Douillard, J. M. & Henry, M. Calculation of surface enthalpy of solids from an ab initio electronegativity based model: case of ice. *J. Colloid Interface Sci.* **263**, 554-561 (2003).

15 Pan, D. *et al.* Surface energy and surface proton order of the ice Ih basal and prism surfaces. *J. Phys.: Condens. Matter* **22**, 074209 (2010).

16 Wei, P., Zhuang, D., Zheng, Y.-Y., Zaoui, A. & Ma, W. Temperature and pressure effect on tensile behavior of ice-Ih under low strain rate: A molecular dynamics study. *J. Mol. Liq.* **355**, 118945 (2022).

17 Sinha, N. K. in *Sixth International Offshore Mechanics and Arctic Engineering Symposium* Vol. 4 189-195 (Houston, Texas, USA, 1987).

18 Ambrožič, B. *et al.* Controlling the radical-induced redox chemistry inside a liquid-cell TEM. *Chem. Sci.* **10**, 8735-8743 (2019).

19 Pimblott, S. M., LaVerne, J. A., Mozumder, A. & Green, N. J. B. Structure of electron tracks in water. 1. Distribution of energy deposition events. *J. Phys. Chem.* **94**, 488-495 (1990).

20 Yesibolati, M. N. *et al.* Electron inelastic mean free path in water. *Nanoscale* **12**, 20649-20657 (2020).

21 Pastina, B. & LaVerne, J. A. Effect of Molecular Hydrogen on Hydrogen Peroxide in Water Radiolysis. *J. Phys. Chem. A* **105**, 9316-9322 (2001).

22 Abellan, P., Gautron, E. & LaVerne, J. A. Radiolysis of Thin Water Ice in Electron Microscopy. *J. Phys. Chem. C* **127**, 15336-15345 (2023).
