# Supplementary material for: Molecular-resolution imaging of ice crystallized from liquid water by cryogenic liquid-cell TEM
Source: Nat Commun. 2025 Sep 25;16:8342. doi: 10.1038/s41467-025-62451-0 (PMC12462463; doi:10.1038/s41467-025-62451-0)
Supplement: Supplementary file 2 — Description of Additional Supplementary Files [file 41467_2025_62451_MOESM2_ESM.pdf]

Description of Additional Supplementary Files

File Name: Supplementary Movie 1
Description: Time-series HRTEM images of an ice crystal along the [0001] zone axis under an electron flux of 60 e $Å^{-2}$ s^{-1}. Interval: 10 s. Duration: 10 min.

File Name: Supplementary Movie 2
Description: Time-series HRTEM images of an ice crystal along the [0001] zone axis under an electron flux of 100 e $Å^{-2}$ s^{-1}. Interval: 10 s. Duration: 10 min.

File Name: Supplementary Movie 3
Description: Focal-series HRTEM images of a through-hole in thin ice films (left) and corresponding Fourier transform (right).

File Name: Supplementary Movie 4
Description: Time-sequence HRTEM images (left), lowpass-filtered HRTEM images (middle), and Fourier transform (right) of bubble nucleation and growth.

File Name: Supplementary Movie 5
Description: Time-sequence HRTEM images (left), lowpass-filtered HRTEM images (middle), and Fourier transform (right) of bubble dissolution.

File Name: Supplementary Movie 6
Description: Time-sequence HRTEM images (left), lowpass-filtered HRTEM images (middle), and Fourier transform (right) of bubble coalescence.

File Name: Supplementary Data 1
Description: Models from MD simulations of ice grain boundaries with various tilt angles and thicknesses. Format: coordinate files, xyz.

File Name: Supplementary Data 2
Description: Models from MD simulations of ice cavities. Format: coordinate files, xyz.

File Name: Supplementary Data 3
Description: Temperature-dependent concentration evolution of chemical species from radiolysis calculations. Format: Microsoft Excel worksheets, xlsx.
